# SPOP mediates apoptosis and protects against necroptosis by regulating ubiquitination of RIPK1 and RIPK3

**DOI:** 10.1172/jci.insight.180655

**Published:** 2025-10-22

**Authors:** Yuzhong Ye, Changjie Yue, Zaosong Zheng, Hailong Ruan, Yuanpeng Zhang, Qi Miao, Xiaoping Zhang, Wen Xiao, Lei Liu

**Affiliations:** 1 Department of Urology, Union Hospital, Tongji Medical College, Huazhong University of Science and Technology, Wuhan, China.; 2 Department of Urology, Fujian Medical University Union Hospital, Fuzhou, China.; 3 Department of Urology, Traditional Chinese and Western Medicine Hospital of Wuhan, Tongji Medical College, Huazhong University of Science and Technology, Wuhan, China.; 4 Department of Urology, Nanfang Hospital, Southern Medical University, Guangzhou, China.; 5 Shenzhen Huazhong University of Science and Technology Research Institute, Shenzhen, China.

**Keywords:** Cell biology, Oncology, Molecular biology

## Abstract

Apoptosis and necroptosis are 2 distinct destinies of cells stimulated with TNF-α; however, it remains unclear how apoptosis and necroptosis are differentially regulated. This study validates the key regulatory role of speckle-type POZ protein (SPOP) in balancing apoptosis and necroptosis. SPOP promotes the polyubiquitination and degradation of receptor-interacting serine/threonine-protein kinase 3 (RIPK3), thereby inhibiting necrosome formation and decreasing cellular sensitivity to necroptosis. Conversely, SPOP interacted with RIPK1 independently of its E3 ubiquitin ligase activity, protecting it from ubiquitination and degradation, thereby enhancing RIPK1 expression and cellular sensitivity to apoptosis. Inhibiting RIPK1 kinase activity with 7-Cl-O-Nec-1 impeded both SPOP-mediated apoptosis and SPOP deficiency–mediated necroptosis. Besides, inhibition or loss of RIPK3 rescued SPOP deficiency–mediated necroptosis. Pancancer analyses indicated that the SPOP/RIPK1/RIPK3 axis is dysfunctional in a variety of tumors. In 3 representative tumor types with high expression of SPOP and RIPK1, kidney renal clear cell carcinoma, liver hepatocellular carcinoma, and breast invasive carcinoma, this regulatory mechanism remains applicable. Based on these findings, a combination therapy using the second mitochondria-derived activator of caspases (Smac) mimetic SM164 and sunitinib was developed, demonstrating a more pronounced efficacy than sunitinib monotherapy, and this sensitizing effect was dependent on the expression level of RIPK1. These results suggest that the combination of Smac mimetics with tyrosine kinase inhibitors holds potential clinical value for tumors with dysregulated SPOP/RIPK1/RIPK3 signaling.

## Abstract 2

SPOP mediates apoptosis and protects against necroptosis. SPOP interacts with RIPK1/RIPK3 and influences their protein stability. Sunitinib and SM164 cotreatment induces RIPK1-dependent cell death in tomors.

## Introduction

Regulated cell death (RCD) is an intricate set of signaling cascades involved in various human diseases, such as cancers and Alzheimer’s disease, and has shown great potential in tumor-targeted therapy (1, 2). Necroptosis and apoptosis are 2 important RCD mechanisms, both inducible by tumor necrosis factor-α (TNF-α) (3). After TNF receptor 1 (TNFR1) is activated by TNF-α, it rapidly recruits cellular inhibitor of apoptosis protein 1/2 (cIAP1/2), receptor-interacting serine/threonine-protein kinase 1 (RIPK1), TNF receptor-associated factor 2 (TRAF2), and TNFR1-associated death domain protein (TRADD) to form TNFR1 signaling complex (TNF-RSC). In different cell contexts, TNF-RSC can subsequently direct cells to distinct fates. In the presence of survival signals, cIAP1/2 mediate the ubiquitination of RIPK1 for further activating of transforming growth factor beta-activated kinase 1 (TAK1) and forming of inhibitor of κB kinase (IKK) complex to activate NF-κB (4, 5). In the absence of survival signaling, TNF-RSC leads to apoptosis (6). Apoptosis is mediated by the activation of caspases, subsequently breaking down cellular components by regulated proteolysis and execution of apoptotic cell death in both RIPK1 kinase–dependent and –independent manners (7, 8). If receptor-interacting serine/threonine-protein kinase 3 (RIPK3) is expressed while preventing apoptosis by inhibiting caspase-8, necroptosis can be triggered and is characterized by the formation of RIPK1/RIPK3 complex (necrosome) and phosphorylated mixed lineage kinase domain-like protein (p-MLKL) (9, 10). Heretofore, it remains largely unclear how apoptosis and necroptosis might be differently regulated under physiological and pathological conditions in vitro and in vivo.

Given the critical and widespread role of RCD in various human diseases, a large number of small-molecule compounds targeting RCD have been screened and developed in recent years (11, 12). Among these, second mitochondria-derived activator of caspases (Smac) mimetics have garnered great attention as a promising class of therapeutic agents (13). Smac is an endogenous pro-apoptotic protein that primarily promotes apoptosis by antagonizing inhibitor of apoptosis proteins (IAPs), which relieves their suppression of caspases and enhances caspase catalytic activity. SM164 is a representative small-molecule Smac mimetic that induces RIPK1-dependent apoptosis by neutralizing IAPs (14). However, in the presence of caspase inhibitors, SM164 can activate necroptosis (15). In tumor cells, aberrant overexpression of IAPs is often associated with evasion of apoptosis and the development of therapeutic resistance. As a result, targeting IAPs to promote apoptosis has become an important strategy in cancer therapy. Most Smac mimetics are still at the preclinical stage, and how to translate these agents into effective clinical therapies remains a great challenge (13, 16).

BTB domain-containing speckle-type POZ protein (SPOP) is a Cullin3-based E3 ubiquitin ligase adaptor protein that can participate in the ubiquitination and degradation of various proteins, such as pancreatic and duodenal homeobox 1, GLI family zinc finger 2, and GLI family zinc finger 3 (17–19). The specific recognition substrate of SPOP is closely related to the MATH domain (20). Recent studies have shown that SPOP plays vital roles in the tumorigenesis of various tumors, such as prostate cancer, renal cell carcinoma (RCC), and colorectal cancer (21–24). However, the involvement of SPOP in TNF-α–mediated cell death has not been reported, and the potential use of SPOP-related mechanisms in antitumor therapy remains unexplored.

Advanced and metastatic tumors are primarily treated with systemic therapies, among which tyrosine kinase inhibitors (TKIs) represent one of the most widely used classes of targeted drugs (25, 26). Sunitinib is a representative TKI, which inhibits multiple tyrosine kinase targets, such as VEGFR, PDGFR, and fms-like tyrosine kinase 3 (27). Sunitinib has been approved for the treatment of advanced RCC, gastrointestinal stromal tumors resistant to imatinib, and progressive pancreatic neuroendocrine tumors (28). Preclinical studies have also demonstrated sunitinib’s antitumor effects in liver, breast, and thyroid cancer cells (29–31), though it has not been successfully applied to these cancers. However, TKI resistance limits the long-term efficacy of these drugs (32). To overcome this resistance, combination therapies involving TKIs and immune checkpoint inhibitors have been explored, but only a small subset of patients benefit (33, 34). Therefore, identifying more strategies to overcome TKI resistance remains an urgent priority. Some researchers suggest that targeting apoptosis and necroptosis pathways could be a promising approach to overcoming tumor treatment resistance (35, 36).

This research reported that SPOP, as a key regulator of the TNF-α–mediated RCD pathway, differentially regulates apoptosis and necroptosis. SPOP mediates RIPK3 ubiquitination and degradation, inhibiting necrosome formation and thereby preventing necrosis. Additionally, SPOP interacts with RIPK1 independently of its E3 ligase activity, protecting RIPK1 from ubiquitination and degradation, thus increasing RIPK1 expression and sensitizing cells to RIPK1-dependent apoptosis. Moreover, pancancer analysis has shown that the SPOP/RIPK1/RIPK3 axis is dysregulated in various tumors. Based on this, we developed a combination therapy of SM164 and sunitinib for tumor types with high expression of SPOP/RIPK1. In vivo and in vitro experiments confirmed that the combined therapy significantly outperformed sunitinib alone, which indicates that the clinical potential of combining TKI with Smac mimetics in SPOP/RIPK1/RIPK3 dysfunction tumors represented by RCC.

## Results

### SPOP mediates apoptosis and protects against necroptosis.

**Figure 1:**
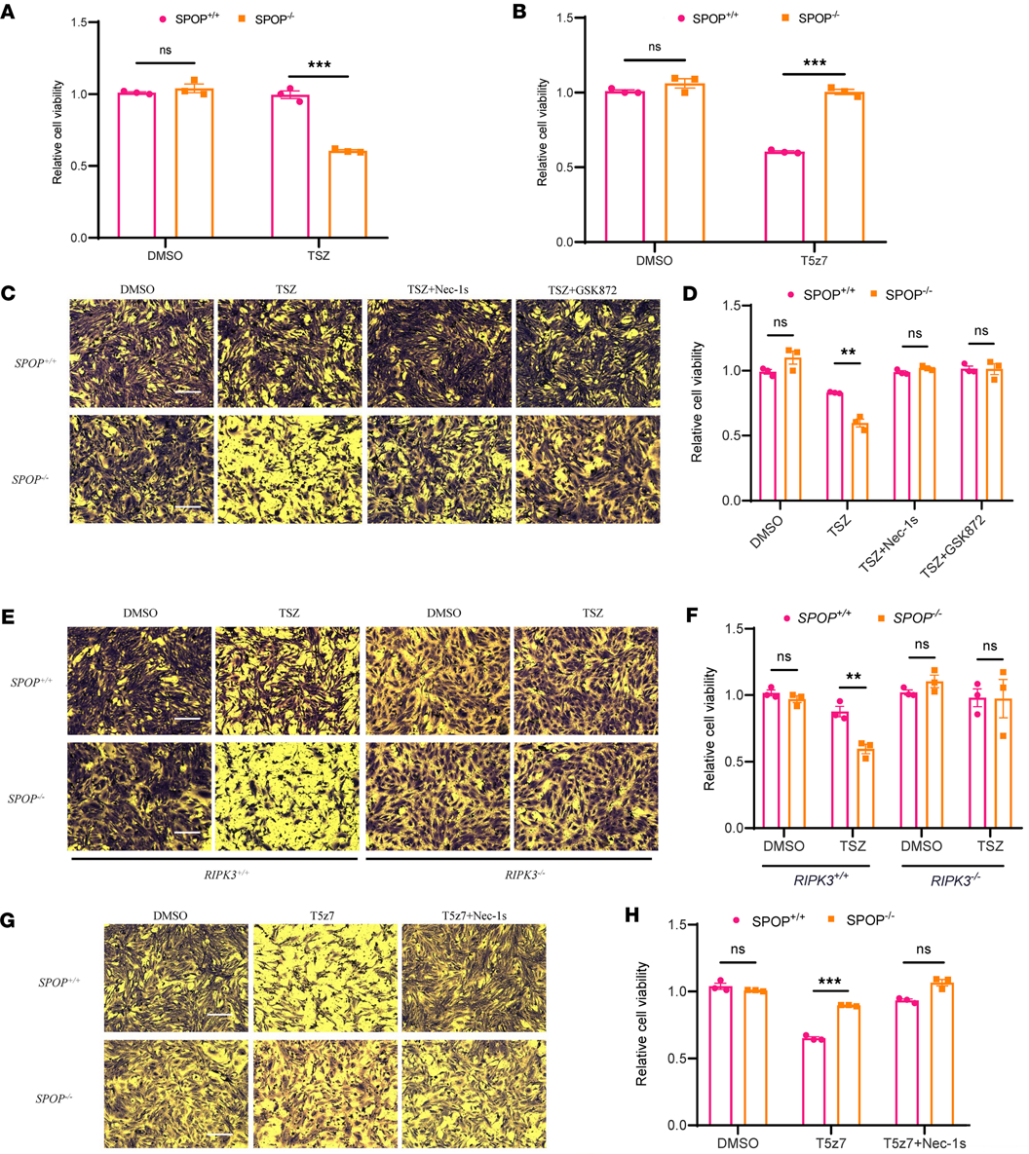
SPOP deficiency protects against apoptosis and mediates necroptosis. (**A**) *SPOP^+/+^* and *SPOP^–/–^* MEFs were treated with DMSO or TSZ for 4 hours and then treated with MTS for 2 hours; subsequently we measured absorbance with fluorescent plate reader. (**B**) *SPOP^+/+^* and *SPOP^–/–^* MEFs were treated with DMSO or T5z7 for 4 hours and then treated with MTS for 2 hours; subsequently we measured absorbance with fluorescent plate reader. (**C**) Images of *SPOP^+/+^* and *SPOP^–/–^* MEFs pretreated with DMSO or Nec-1s or GSK872 for 1 hour and then treated with DMSO or TSZ or TSZ + Nec-1s or TSZ + GSK872 for 4 hours. Scale bar, 100 μm. (**D**) *SPOP^+/+^* and *SPOP^–/–^* MEFs were pretreated with DMSO or Nec-1s or GSK872 for 1 hour and then treated with DMSO or TSZ or TSZ + Nec-1s or TSZ + GSK872 for 4 hours and then treated with MTS for 2 hours; subsequently we measured absorbance with fluorescent plate reader. (**E**) Images of *SPOP^+/+^RIPK3^+/+^*, *SPOP^+/+^RIPK3^–/–^*, *SPOP^–/–^RIPK3^+/+^*, and *SPOP^–/–^RIPK3^–/–^* MEFs treated with DMSO or TSZ or 4 hours. Scale bar, 100 μm. (**F**) *SPOP^+/+^RIPK3^+/+^*, *SPOP^+/+^RIPK3^–/–^*, *SPOP^–/–^RIPK3^+/+^*, and *SPOP^–/–^RIPK3^–/–^* MEFs treated with DMSO or TSZ or 4 hours and then treated with MTS for 2 hours; subsequently we measured absorbance with fluorescent plate reader. (**G**) Images of *SPOP^+/+^* and *SPOP^–/–^* MEFs pretreated with DMSO or Nec-1s for 1 hour and then treated with DMSO or T5z7 or T5z7 + Nec-1s for 6 hours. Scale bar, 100 μm. (**H**) *SPOP^+/+^* and *SPOP^–/–^* MEFs pretreated with DMSO or Nec-1s for 1 hour and treated with DMSO or T5z7 or T5z7 + Nec-1s for 6 hours, then treated with MTS for 2 hours; subsequently we measured absorbance with fluorescent plate reader. Experiments were performed in triplicate and repeated 3 times independently. Data are presented as mean ± SD. The significance of differences was revealed based on unpaired Student’s *t* test (***P < 0.001, **P < 0.01).

To validate the role that SPOP plays in RCD, we tested the sensitivity of *SPOP^+/+^* and *SPOP^–/–^* mouse embryonic fibroblasts (MEFs) to apoptosis and necroptosis. Upon SPOP knockout, MEFs became more susceptible to necroptosis induced by TNF-α + SM164 + zVAD.fmk (TSZ) (Figure 1A) and more resistant to apoptosis induced by TNF-α + 5Z-7-Oxozeaenol (T5z7) (Figure 1B). In *SPOP^–/–^* MEFs, the sensitivity to necroptosis could be blocked by RIPK3 inhibitor GSK872, RIPK1 inhibitor 7-Cl-O-Nec-1 (Nec-1s), as well as RIPK3 knockout (Figure 1, C–F). Notably, in *SPOP^+/+^* MEFs, the sensitivity to apoptosis could be also rescued by inhibition of RIPK1 (Figure 1, G and H, and Supplemental Figure 1; supplemental material available online with this article; https://doi.org/10.1172/jci.insight.180655DS1). Together, these findings suggest that SPOP prevents necroptosis and mediating apoptosis.

### SPOP regulates apoptosis and necroptosis through RIPK1 and RIPK3.

**Figure 2:**
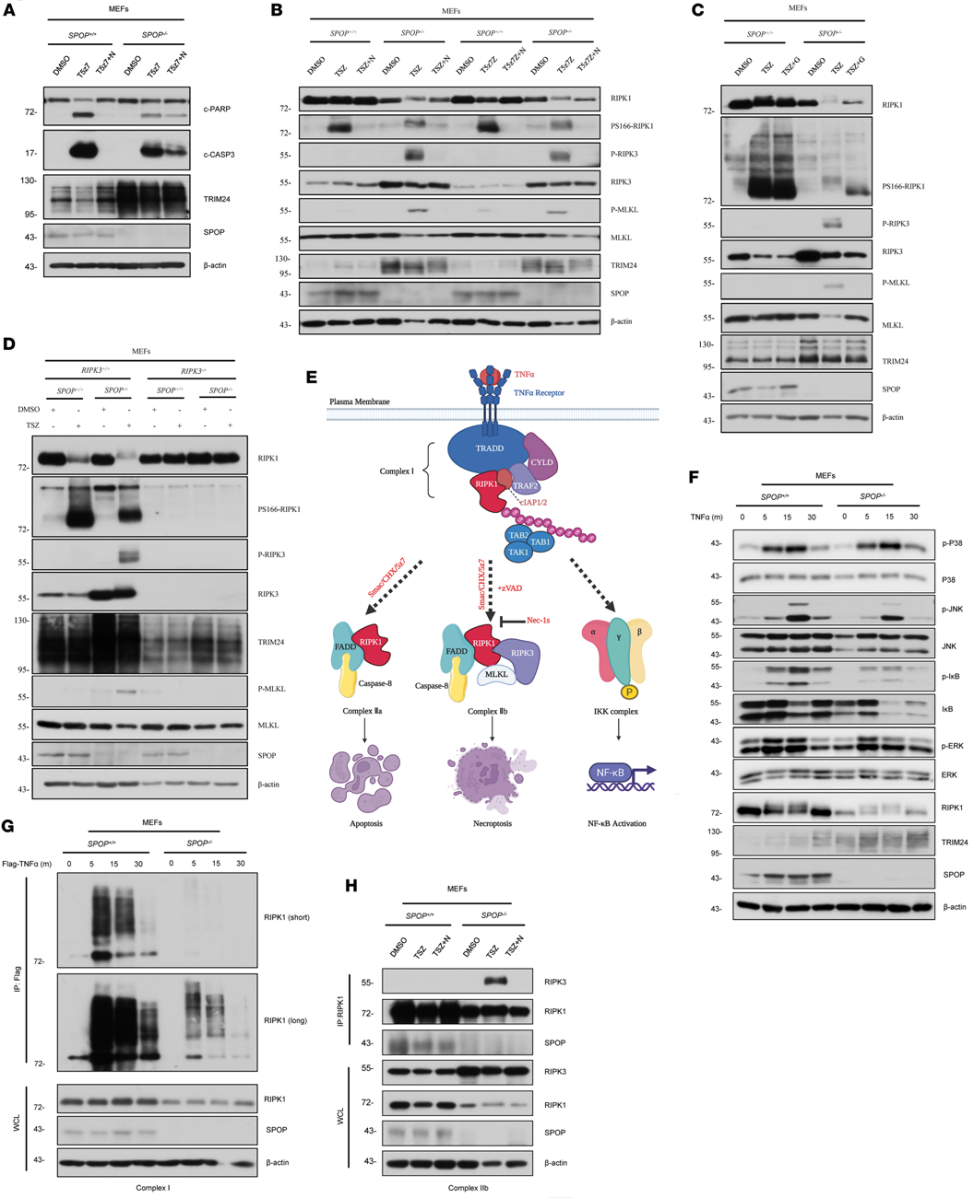
SPOP deficiency breaks the balance between RIPK1 and RIPK3 and facilitates the formation of necrosome. (**A**) *SPOP^+/+^* and *SPOP^–/–^* MEFs were pretreated with Nec-1s or DMSO for 1 hour and then treated with DMSO or T5z7 or T5z7+N for 6 hours; subsequently whole cell lysate (WCL) was harvested for immunoblot (IB) analysis. Values on left are in kilodaltons. C-, cleaved. (**B**) *SPOP^+/+^* and *SPOP^–/–^* MEFs were pretreated with Nec-1s (N) or DMSO for 1 hour and then treated with DMSO or TSZ or TSZ+N or T5z7Z or T5z7Z+N for 4 hours; subsequently WCL was harvested for IB analysis. (**C**) *SPOP^+/+^* and *SPOP^–/–^* MEFs were pretreated with GSK872 (G) or DMSO for 1 hour and then treated with DMSO or TSZ or TSZ+G for 4 hours; subsequently WCL was harvested for IB analysis. (**D**) IB analysis of WCL derived from *RIPK3^+/+^* and *RIPK3^–/–^* MEFs with SPOP knockout through CRISPR technology. Parental MEFs were used as the control. Cells were treated with TSZ or DMSO for 4 hours; subsequently WCL was harvested for IB analysis. (**E**) A schematic illustration of RIPK1-mediated multimodal signaling events downstream of TNFR1. (**F**) *SPOP^+/+^* and *SPOP^–/–^* MEFs were stimulated by TNF-α for the indicated periods of time. WCL was harvested for IB analysis. (**G**) *SPOP^+/+^* and *SPOP^–/–^* MEFs were stimulated by FLAG–TNF-α for the indicated periods of time, and TNF-RSC was immunoprecipitated (IP) using anti-Flag resin and analyzed using the indicated antibodies. (**H**) *SPOP^+/+^* and *SPOP^–/–^* MEFs were pretreated with Nec-1s or DMSO for 1 hour and then treated with DMSO or TSZ or TSZ+N for 4 hours. Necrosome was immunoprecipitated using RIPK1 antibody and analyzed using the indicated antibodies.

To further explore the mechanism by which SPOP regulates RCD, we examined markers of apoptosis and necroptosis. In *SPOP^+/+^* MEFs stimulated by T5z7 to induce apoptosis, the apoptosis marker cleaved poly (ADP-ribose) polymerase (PARP) and cleaved caspase-3 were much stronger than in *SPOP^–/–^* MEFs, which could be partially rescued by the combination usage of Nec-1s (Figure 2A). In *SPOP^–/–^* MEFs stimulated by TSZ and T5z7 + zVAD.fmk (T5z7Z) to induce necroptosis, increased phosphorylation of S345 MLKL and S232 RIPK3 (2 markers for necroptosis) was observed, while a clear bandshift of the phosphorylation of S166 RIPK1 was also observed (Figure 2B). Furthermore, the increased phosphorylation of S345 MLKL and S232 RIPK3 could be blocked by inhibition of RIPK1, inhibition of RIPK3, and loss of RIPK3, and the bandshift of PS166 RIPK1 was reversed by Nec-1s (inhibitor of RIPK1) or GSK872 (inhibitor of RIPK3) (Figure 2, B–D). Taken together, SPOP affects apoptosis and necroptosis by influencing the RIPK1/RIPK3/MLKL axis.

### SPOP controls necrosome formation to prevent necroptosis.

As previously described, following TNF-α/TNFR1 activation, cells have 3 destinies: apoptosis, necroptosis, or survival via NF-κB activation (Figure 2E). To examine downstream molecular events and protein interactions, we stimulated *SPOP^+/+^* and *SPOP^–/–^* MEFs with TNF-α or Flag–TNF-α in a time-dependent manner. We found that SPOP deficiency markedly decreased the recruitment of ubiquitinated RIPK1 within TNF-RSC (Figure 2G), but SPOP deficiency resulted in no obvious changes in NF-κB signaling or other survival-related signaling molecules (p38/p-p38, JNK/p-JNK, and ERK/p-ERK) (Figure 2F). In contrast, SPOP deficiency increased RIPK3 protein levels and enhanced the interaction between RIPK1 and RIPK3, leading to necrosome formation, which could be impeded by inhibition of RIPK1 (Figure 2H). These findings suggest that SPOP prevents necroptosis by inhibiting the interaction between RIPK1 and RIPK3, thereby blocking necrosome formation.

### SPOP doubly regulates the ubiquitination level of RIPK3 and RIPK1.

**Figure 3:**
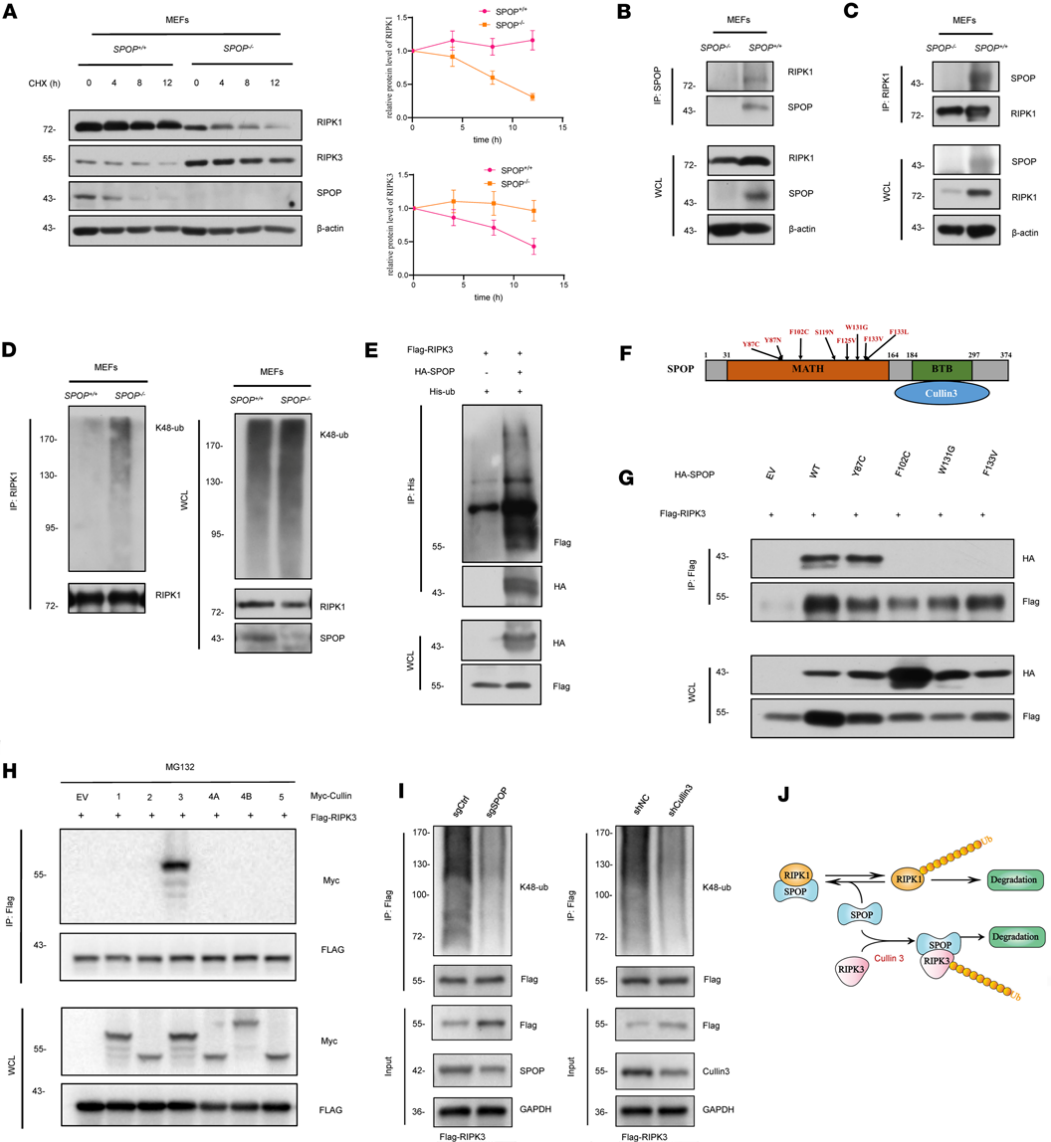
SPOP interacts with RIPK1/RIPK3 and influences their ubiquitination. (**A**) IB analysis of WCL derived from *SPOP^+/+^* and *SPOP^–/–^* MEFs, as well as corresponding quantitative analysis. Cells were treated with 1 μM cyclohexamide (CHX) for the indicated time period before harvesting. Experiments repeated 3 times independently and data are presented as mean ± SD. (**B** and **C**) IB analysis of co-IP and WCL derived from *SPOP^+/+^* and *SPOP^–/–^* MEFs to show that endogenous RIPK1 interacts with endogenous SPOP. Cells were treated with 20 μM MG132 for 4 hours before harvesting. (**D**) Ubiquitination (ub) assay was performed using RIPK1 antibody in *SPOP^+/+^* and *SPOP^–/–^* MEFs. Cells were treated with 10 μM MG132 for 4 hours before harvesting. (**E**) IB analysis of WCL and His tag pull-down products derived from HEK293T cells transfected with indicated plasmids. Cells were treated with 20 μM MG132 for 6 hours before harvesting. (**F**) Schematic of SPOP domains and prostate-associated mutations that failed to interact with substrates as literature reported. (**G**) IB analysis of co-IP and WCL derived from HEK293T transfected with Flag-RIPK3 and indicated HA-SPOP. Cells were treated with 20 μM MG132 for 6 hours before harvesting. (**H**) IB analysis of co-IP and WCL derived from HEK293T transfected with Flag-RIPK3 and Myc-Cullin. Cells were treated with 20 μM MG132 for 6 hours before harvesting. (**I**) IB analysis of co-IP and WCL derived from HEK293T with overexpressed RIPK3 transfected with Cullin3 shRNA (shCullin3) or sgSPOP. (**J**) A model for the role of SPOP in necroptosis and apoptosis.

To investigate the mechanism by which SPOP influences the protein level of RIPK1 and RIPK3, protein stability of RIPK1 and RIPK3 was detected in *SPOP^+/+^* and *SPOP^–/–^* MEFs. Compared with *SPOP^+/+^* MEFs, the half-life of RIPK1 was noticeably reduced in *SPOP^–/–^* MEFs, while the half-life of RIPK3 was noticeably elevated (Figure 3A), suggesting that SPOP might control the expression of RIPK1/RIPK3 largely through a posttranslational mechanism. We performed molecular docking analysis of the interaction between SPOP and RIPK1 or RIPK3, and the results revealed that SPOP contains specific protein sites capable of directly binding to RIPK1 or RIPK3 (Supplemental Figure 2). Furthermore, the results of co-immunoprecipitation (co-IP) showed that RIPK1 and SPOP interacted with each other in *SPOP^+/+^* MEFs (Figure 3, B and C). As mentioned above, the classic function of SPOP is to act as an E3 ubiquitin ligase adaptor protein and play an important role in the ubiquitination and degradation of many proteins. We explored the ubiquitination level of RIPK1 in *SPOP^+/+^* and *SPOP^–/–^* MEFs, verifying that more RIPK1 was ubiquitinated in *SPOP^–/–^* MEFs (Figure 3D). These data suggest that SPOP interacts with RIPK1 and protects RIPK1 from polyubiquitination.

Since there is no good commercial RIPK3 antibody for co-IP, we used HEK293T transfected with indicated RIPK3, SPOP, and ubiquitin plasmids to explore the ubiquitination of RIPK3, and we found that SPOP overexpression could induce polyubiquitination of RIPK3 (Figure 3E). The MATH domain of SPOP is its substrate-binding domain, and multiple mutations (Y87C, F102C, W131G, F133V) can directly affect its function (Figure 3F). Next, we explored whether SPOP mutations within MATH domain could affect the interaction between SPOP and RIPK3. RIPK3 was identified to interact with WT SPOP, where most of the MATH domain mutation failed to interact with RIPK3 (Figure 3G) (18). The ubiquitin ligase Cullin family members are scaffold proteins of the E3 ubiquitin ligase complex, which recruits substrates for ubiquitination modification by binding to adaptor proteins. RIPK3 and different Cullin family members were transfected into HEK293T cells, and co-IP results showed that RIPK3 bound to Cullin3 but not to Cullin1, 2, 4A, 4B, and 5 (Figure 3H). Moreover, knocking down Cullin3 or SPOP led to upregulation of RIPK3 expression and decreased the ubiquitination of RIPK3 (Figure 3I). Thus, we consider SPOP an E3 ligase adaptor protein that might target RIPK1/RIPK3 for regulating their protein stability, respectively, in a ubiquitination activity–independent and –dependent manner (Figure 3J).

### SPOP/RIPK1/RIPK3 is dysfunctional in a variety of tumors.

**Figure 4:**
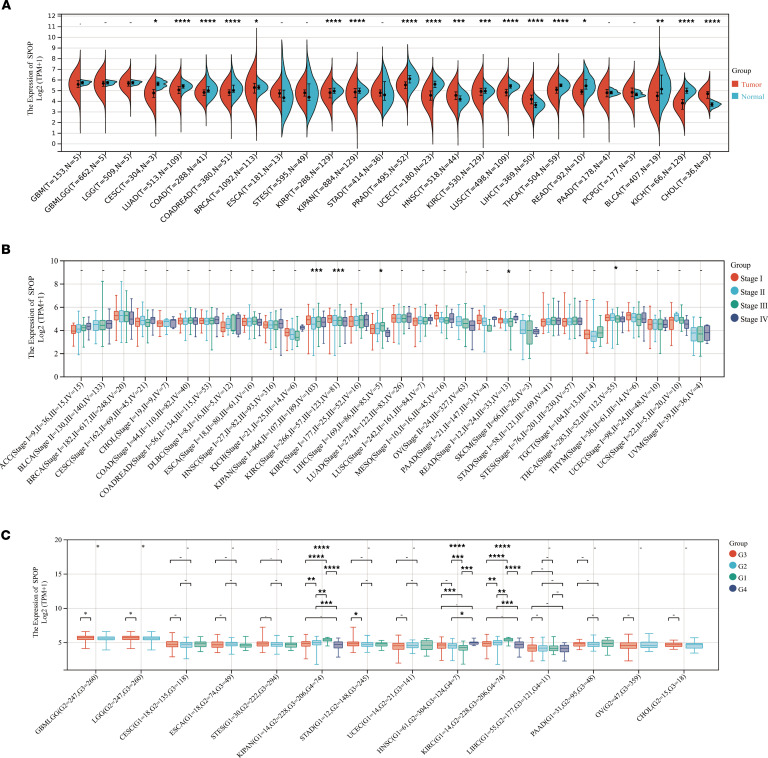
Pancancer analysis of SPOP. (**A**) Pancancer analysis of SPOP expression in 26 kinds of tumors and corresponding normal tissues after procedural exclusion. TPM, transcripts per million. (**B** and **C**) Pancancer analysis of SPOP expression among different clinical stages in 30 kinds of tumors and different pathological grades in 14 kinds of tumors after procedural exclusion. Data are presented as mean ± SD. The significance of differences was revealed based on unpaired Student’s *t* test for 2 independent groups, Wilcoxon rank sum and signed rank tests for 2 groups in multiple samples, and Kruskal test for multiple groups (****P < 0.001, ***P < 0.001, **P < 0.01, *P < 0.05).

**Figure 5:**
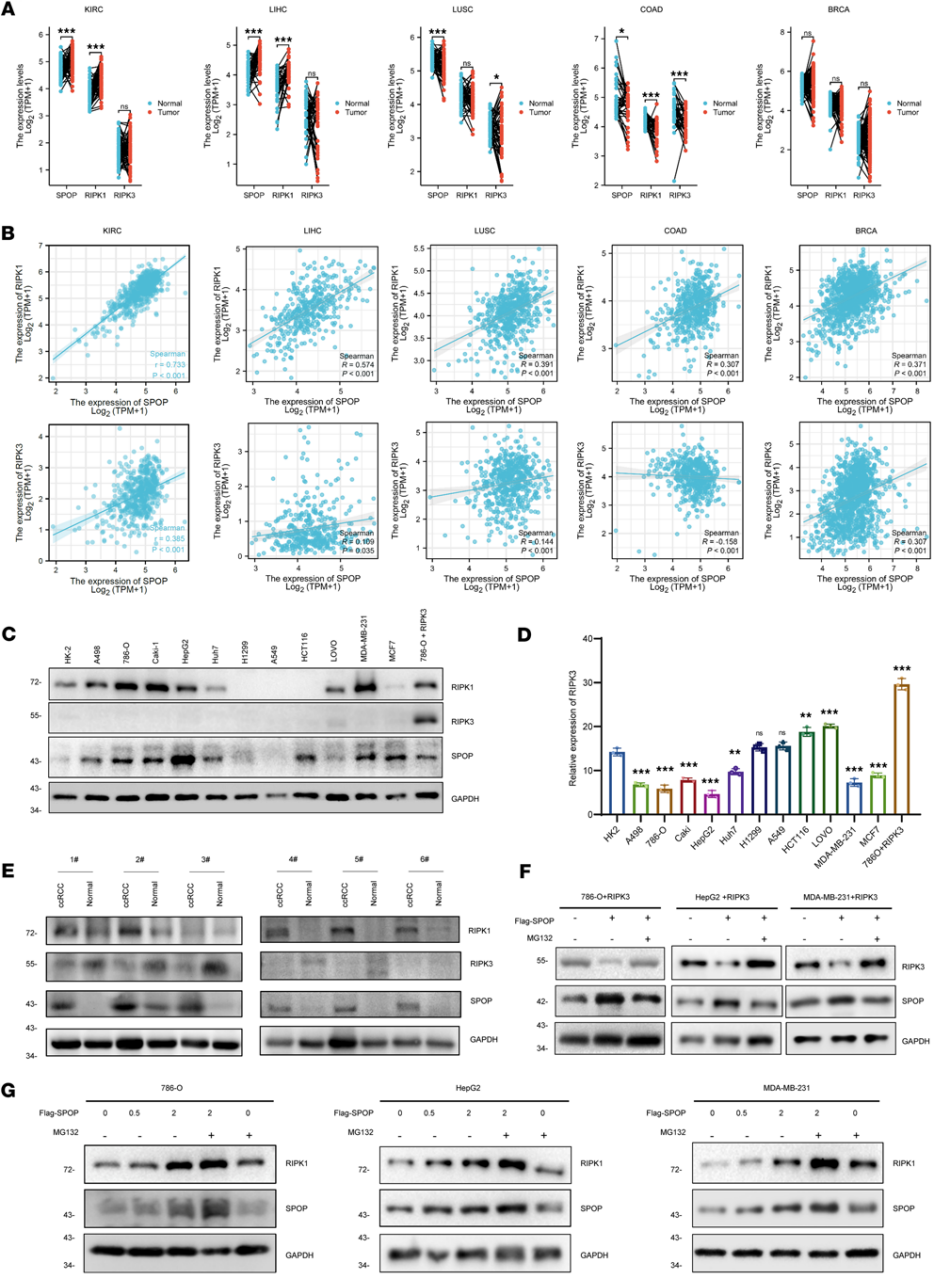
SPOP correlates with RIPK1 rather than RIPK3 in tumors. (**A**) The mRNA expression level of RIPK1, RIPK3, and SPOP between tumor tissues and adjacent normal tissues in TCGA. (**B**) Correlation analysis between SPOP and RIPK1/RIPK3 in tumors. (**C**) IB analysis of the protein expression level of RIPK1, RIPK3, and SPOP in different tumor cell lines. (**D**) The mRNA level of RIPK3 in different tumor cell lines. (**E**) IB analysis of the protein expression level of RIPK1, RIPK3, and SPOP in RCC tissue samples. (**F**) IB analysis of the RIPK1/RIPK3 protein level exchange derived from 786-O+RIPK3, HepG2+RIPK3, and MDA-MB-231+RIPK3 transfected with indicated Flag-SPOP. (**G**) IB analysis of the RIPK1 protein level exchange derived from 786-O, HepG2, and MDA-MB-231 transfected with indicated Flag-SPOP. Cells were treated with 20 μM DMSO or MG132 for 6 hours before harvesting. Results represent at least 3 independent experiments. The significance of differences was revealed based on Student’s *t* test for 2 unpaired or paired groups. The correlation analysis was conducted using Pearson correlation coefficient (***P < 0.001, **P < 0.01, *P < 0.05).

Given that cancer is often characterized by an imbalance between apoptosis and necroptosis (37, 38), we investigated the role of the SPOP/RIPK1/RIPK3 axis in tumors. First, we conducted a pancancer exploration using data from The Cancer Genome Atlas (TCGA) (33). SPOP expression was significantly upregulated in 15 of the 34 tumor types, including kidney renal clear cell carcinoma (KIRC), head and neck squamous cell carcinoma (HNSC), liver hepatocellular carcinoma (LIHC), and breast invasive carcinoma (BRCA), and significantly downregulated in 14 tumor types, such as lung squamous cell carcinoma (LUSC), lung adenocarcinoma (LUAD), and colon adenocarcinoma (COAD) (Figure 4A). We further examined *SPOP* expression across clinical stages and pathological grades and observed significant differences only in KIRC among the 26 tumor types (Figure 4, B and C). The pancancer analysis results of *RIPK1* and *RIPK3* also revealed that the protein levels of *RIPK1* and *RIPK3* were dysregulated in most tumors. Additionally, *RIPK1* expression in KIRC was significantly associated with clinical and pathological stages (Supplemental Figures 3 and 4). Therefore, we selected 5 types of tumors, KIRC, LIHC, LUAD, COAD, and BRCA, for further analysis. A paired analysis of tumor and adjacent normal tissues in TCGA revealed that *SPOP* expression remained significantly elevated in KIRC and LIHC and significantly decreased in LUSC and COAD (Figure 5A). Correlation analysis showed that *SPOP* and *RIPK1* were positively correlated in all 5 cancer types (Figure 5B). We further assessed the protein levels of SPOP, RIPK1, and RIPK3 in representative cell lines of 5 cancer types. Immunoblotting results showed simultaneous upregulation of SPOP and RIPK1 in KIRC (A498, 786-O, Caki-1), LIHC (Huh7, HepG2), and BRCA (MDA-MB-231) cell lines and simultaneous downregulation in LUSC cell lines (H199, A549). However, similar patterns were not observed in COAD cell lines (HCT116, LOVO) or in the BRCA MCF7 cell line (Figure 5C). RIPK3 protein levels were very low and barely detectable via immunoblotting (Figure 5C), so we detected the mRNA level of *RIPK3*. The mRNA levels of *RIPK3* displayed an expression trend opposite to that of SPOP across the tumor cell lines (Figure 5D). Additionally, we examined protein levels in 6 pairs of KIRC tumor and adjacent normal tissues. SPOP and RIPK1 were substantially overexpressed in tumor tissues, while RIPK3 expression was reduced (Figure 5E). These results suggest that the SPOP/RIPK1/RIPK3 axis is dysregulated in most tumor types.

RIPK3 expression has been previously shown to be regulated via transcriptional repression mechanisms, including promoter hypermethylation (37). This supports the notion that the SPOP specifically regulates RIPK1 protein stability in a cell content–dependent manner, while RIPK3 might escape the regulation of SPOP by transcriptional silencing. We selected 786-O, HepG2, and MDA-MB-231, 3 cancer cell lines with high expression of SPOP and RIPK1, for further verification. Consistent with our previous findings, in these 3 cell lines, overexpressed SPOP increased the expression of RIPK1 in a dose-dependent manner, and the proteasome inhibitor MG132 could further increase RIPK1 level (Figure 5G). On the contrary, SPOP decreased the protein level of RIPK3, which could be blocked by MG132 (Figure 5F). These results indicate that SPOP-mediated regulation of RIPK1 and RIPK3 persists across various tumor types.

### Investigating tumor therapy strategy in tumors with high expression of SPOP and RIPK1.

**Figure 6:**
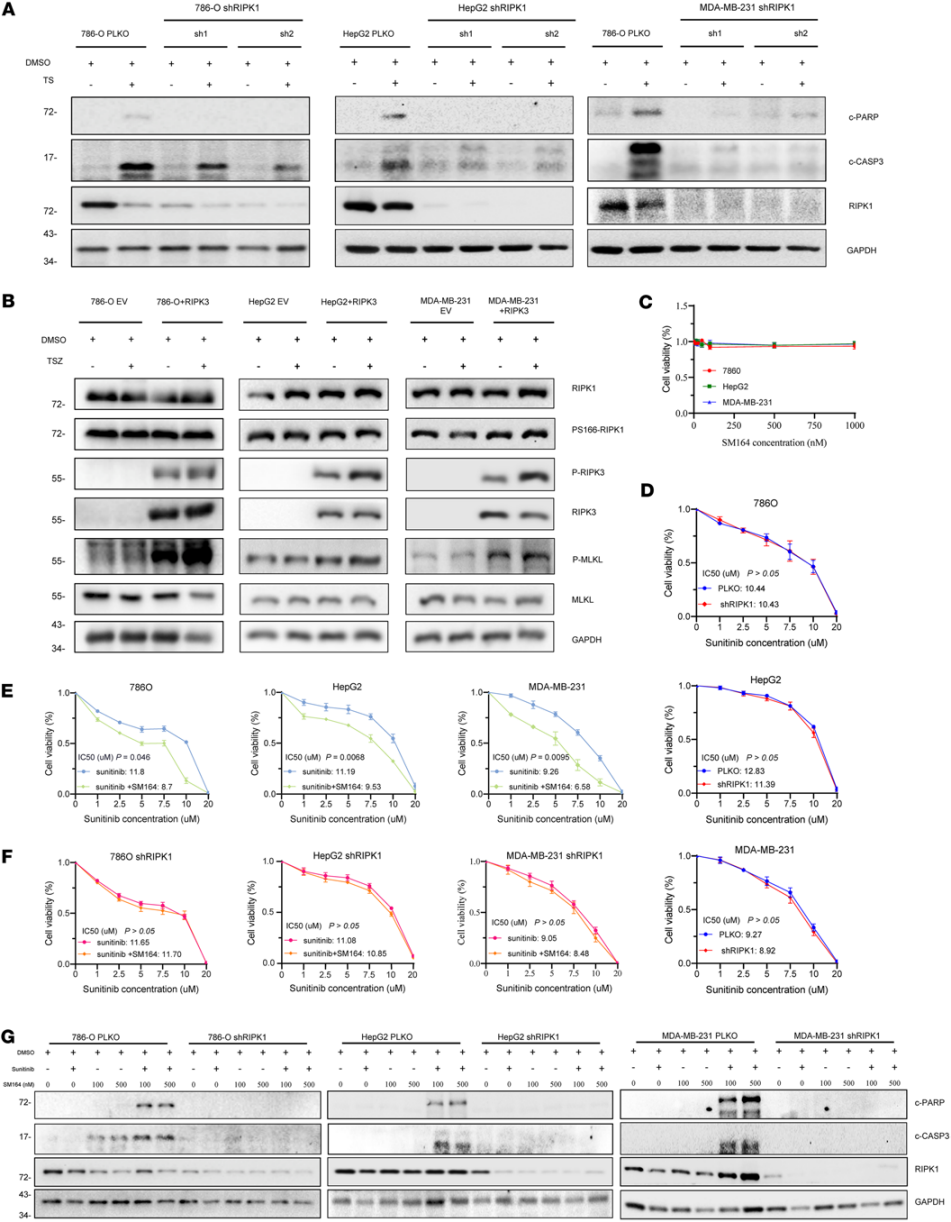
RIPK1 expression is associated with the sensitivity to sunitinib and SM164 cotreatment in tumor. (**A**) After knockdown of RIPK1 in 786-O, HepG2, and MDA-MB-231 cells, the protein levels of apoptotic markers mediated by TNF-α+SM164 (TS) stimulation were detected. (**B**) WT and RIPK3-overexpressing 786-O, HepG2, and MDA-MB-231 cells were treated with DMSO or TSZ for 6 hours; subsequently WCL was harvested for IB analysis. (**C**) Cell viability assay to assess the sensitivity of 786-O, HepG2, and MDA-MB-231 cells to SM164 treatment. (**D**) Cell viability assay for the effect of RIPK1 knockdown on the sensitivity of 786-O, HepG2, and MDA-MB-231 cells to sunitinib treatment. (**E**) Cell viability assay to assess the effect of combined use of SM164 on sunitinib sensitivity in 786-O, HepG2, and MDA-MB-231 cells. (**F**) Cell viability assay for the effect of RIPK1 knockdown on the sensitivity of 786-O, HepG2, and MDA-MB-231 cells to sunitinib alone, and sunitinib combined with SM164 treatment. (**G**) WT and RIPK1-knockdown 786-O, HepG2, and MDA-MB-231 cells were treated with DMSO, sunitinib alone, SM164 alone, or sunitinib combined with SM164 for 24 hours; subsequently WCL was harvested for IB analysis. Cell Counting Kit-8 (CCK-8) experiments were performed in triplicate and repeated 3 times independently. Data are presented as mean ± SD. The significance of differences was revealed based on paired Student’s *t* test.

Although TSZ treatment markedly increased the phosphorylation level of RIPK3 (Figure 6B), the basal expression level of RIPK3 in tumor cell lines was extremely low. In addition, TSZ triple combination is less likely to be achieved in the clinic, so we did not carry out further research about RIPK3 in tumors. Our results showed that SPOP can prevent necroptosis and mediate apoptosis. In theory, inducing apoptosis in tumors with abnormally high expression of SPOP can more easily kill tumor cells. Since there are currently no commercial small-molecule regulators for SPOP (39), we turned to RIPK1 as a potential antitumor drug target.

We silenced RIPK1 in 786-O, HepG2, and MDA-MB-231 using 3 shRNAs. Immunoblotting results of cleaved PARP and cleaved caspase-3 showed that RIPK1 knockdown inhibited apoptosis following TNF-α+SM164 (TS) treatment (Figure 6A). Per the literature, sunitinib can activate the NF-κB signaling pathway of tumor cells and upregulate TNF-α, IL-6, and IL-8 (40). This led us to investigate whether sunitinib combined with SM164 could simulate TS treatment. We first tested the cell viability with a single agent. We found SM164 monotherapy did not affect the cell viability of 786-O, HepG2, and MDA-MB-231 (Figure 6C), and RIPK1 knockdown did not affect sunitinib sensitivity in these cells (Figure 6D). Further experimental results showed that SM164 increased the sunitinib sensitivity of 786-O, HepG2, and MDA-MB-231(Figure 6E), and this enhancement could be blocked by RIPK1 knockdown (Figure 6F). Immunoblotting analysis showed sunitinib and SM164 cotreatment exacerbated apoptosis compared with a single agent, which were abrogated by RIPK1 knockdown (Figure 6G). These results indicate that sunitinib and SM164 cotreatment promotes apoptosis in a RIPK1-dependent manner. Therefore, these results remind us that RIPK1 expression–based therapy displays some clinical value for screening potential beneficiaries.

**Figure 7:**
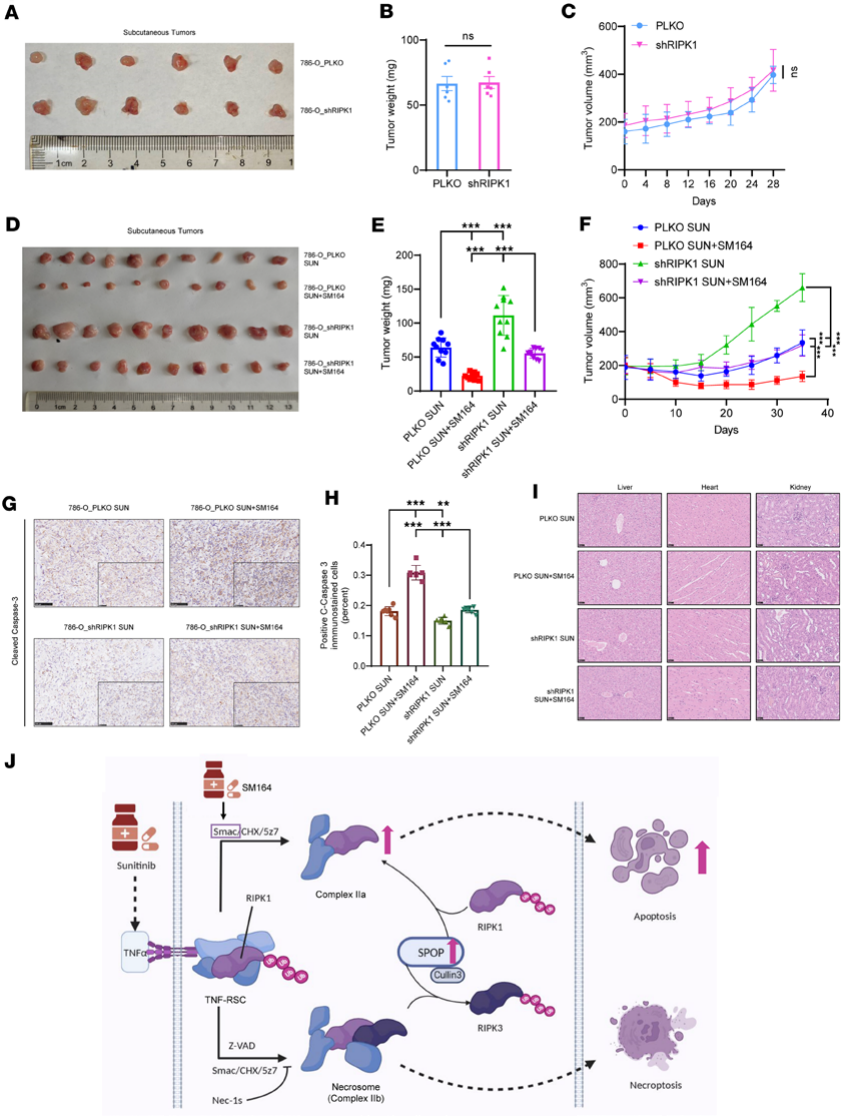
SM164 enhances antitumor activity of sunitinib on xenograft models. (**A**–**C**) Nude mice were injected subcutaneously with 786-O_PLKO or 786-O shRIPK1 cells without treatment. (**A**) Images of isolated tumors. (**B** and **C**) Tumor weights and tumor volume curves of 786-O xenografts (*n* = 6). (**D**–**H**) Nude mice were injected subcutaneously with indicated 786-O cells treated with sunitinib alone or combination of sunitinib and SM164. Sunitinib: 40 mg/kg, orally administered; SM164: 5 mg/kg, intraperitoneal injection. (**D**) Images of isolated tumors. (**E** and **F**) Tumor weights and tumor volume curves of 786-O xenografts in each treatment group (*n* = 10). (**G**) Immunohistochemical analysis of cleaved caspase-3 expression was performed. (**H**) Analysis of positive cleaved caspase-3 (*n* = 7). (**I**) H&E staining tissue images obtained from major organs of xenograft mice for the in vivo toxicity evaluation. Scale bars: 100 µm (**G**), 50 µm (**G** insets), and 50 µm (**I**). (**J**) Schematic diagram illustrated how SM164 and sunitinib promote apoptosis based on SPOP-mediated overexpression of RIPK1. The mean ± SD is shown. Statistical significance was determined using 1-way or 2-way ANOVA with Tukey’s post hoc test. Evaluation of morphology was performed by a board-certified pathologist in a blinded manner. The significance of differences was revealed based on unpaired Student’s *t* test for 2 independent groups and 1-way or 2-way ANOVA with Tukey’s post hoc test for multiple groups (***P < 0.001, **P < 0.01).

Given that sunitinib and SM164 cotreatment effectively induced RIPK1-positive tumor cell death in vitro, their combined effect was then investigated in vivo using a xenograft mouse model. Pancancer analysis suggests that KIRC is the most representative cancer with abnormally high expression of SPOP and RIPK1, so we selected 786-O cells to construct a nude mouse xenograft tumor model. After inoculating 786-O_PLKO/shRIPK1 cells, we observed the growth rate of subcutaneous tumors. No significant difference was found between the 2 groups, indicating that RIPK1 cannot directly affect the growth of KIRC cells in vivo (Figure 7, A–C).

After constructing subcutaneous tumors, we treated nude mice with sunitinib alone or in combination with SM164 for 2 weeks. Compared with sunitinib alone, the combination treatment exhibited increased antitumor activity in a xenograft model, especially in the 786-O_PLKO (RIPK1-positive) group (Figure 7, D–F). Moreover, IHC results showed that cotreatment with SM164 and sunitinib enhanced the expression of cleaved caspase-3, a marker of apoptosis, in tumor tissues, and this effect was attenuated upon RIPK1 knockdown (Figure 7, G and H). In addition, toxicity was not observed in livers, hearts, and kidneys of nude mice (Figure 7I), indicating that the combination treatment is well tolerated in vivo. These results indicate that combination treatment with sunitinib and SM164 can increase antitumor activity in RCC xenograft models.

## Discussion

Utilizing *SPOP^–/–^* MEF cell model, pancancer data analysis, and both in vivo and in vitro tumor models, this study elucidates the role of SPOP in regulating apoptosis and necroptosis and provides more insights for cancer targeted therapy. Under physiological conditions, SPOP can regulate apoptosis and necroptosis by affecting RIPK1 and RIPK3 at the posttranslational modification level. In the context of cancer, SPOP/RIPK1/RIPK3 axis is dysregulated across multiple cancer types, and the combination of the Smac mimetic SM164 enhances the antitumor efficacy of sunitinib in tumors with high expression of SPOP/RIPK1.

RIPK1 and RIPK3 are central proteins in apoptosis and necroptosis, but posttranslational processes that regulate RIPK1 and RIPK3 activity and stability remain poorly understood (41, 42). In this study, we found SPOP binds to RIPK3 via its MATH domain and facilitates RIPK3 ubiquitination and degradation through Cullin3 recruited by its BTB domain, thereby inhibiting necrosome formation and preventing necroptosis. Additionally, SPOP interacts with RIPK1 and suppresses its ubiquitination, leading to elevated RIPK1 levels and increased cellular sensitivity to TNF-α–induced apoptosis (Figure 7J). Liu et al. found that SPOP affects the TNF/JNK signaling pathway through the Drosophila segmentation network model (43). However, we could not detect any interaction between SPOP and TNF-RSC, and data suggested that SPOP did not affect the NF-κB pathway either. Homozygous deletion of SPOP has been shown to cause neonatal lethality (44). Our study showed that SPOP deficiency leads to increased susceptibility of MEFs to necroptosis, which can be blocked by RIPK1 inhibitor and RIPK3 knockout; thus, we wonder if the neonatal lethality by SPOP deletion can be reversed by inactivation of RIPK1 or deletion of RIPK3. Actually, many groups have found that embryo lethality resulting from gene deficiency (such as *caspase8*, *HOIP*, *RIPK1^D161N^*, *FADD*, *Otulin*) could be rescued by *RIPK1^D138N^* or *RIPK3^–/–^* (45–48). These questions would be further studied in future work.

One of the hallmarks of cancer is the evasion of RCD through genetic mutations or epigenetic reprogramming, which leads to uncontrolled tumor cell growth and chemotherapy resistance (49). Pancancer analyses have revealed that the SPOP/RIPK1/RIPK3 signaling axis is dysregulated across various tumors. Studies have shown that SPOP plays a key role in cancers such as RCC, hepatocellular carcinoma, prostate cancer, and lung cancer. In RCC, the aberrantly overexpressed SPOP is positively correlated with tumor metastasis (50). Deng et al. reported that a mutation at the M35L site within the MATH domain of SPOP promotes the proliferation and metastasis of prostate cancer cells (51). In prostate cancer, SPOP acts as a tumor suppressor by facilitating the degradation of multiple oncogenic substrates. However, mutations in its MATH domain enhance the proliferative and invasive capacities of prostate cancer cells and are strongly associated with poor patient prognosis (24). In non–small cell lung cancer (NSCLC), SPOP expression is downregulated and significantly correlated with unfavorable clinical outcomes, suggesting its potential role as a tumor suppressor gene in NSCLC (52). Similarly, reduced SPOP expression in colorectal cancer is significantly associated with adverse clinicopathological features, such as poor differentiation and increased hematogenous metastasis (23). In this study, we also found that in tumors with overexpressed SPOP represented by RCC, RIPK3 might escape the regulation of SPOP by transcriptional silencing. A case-control study of 458 patients with non-Hodgkin lymphoma found that genetic variants in RIPK3 may be important in non-Hodgkin lymphoma development (53); Liu et al. found that chronic lymphocytic leukemia was unable to induce necroptosis by TNF-α/zVAD because of defects in RIPK3 and deubiquitinase cylindromatosis (CYLD), key molecules of the necroptosis signaling cascade (54). The findings of this study may provide a theoretical direction for the development of alternative cancer treatment strategies.

Previous studies have demonstrated that SPOP degrades substrates selectively by recognizing their SPOP-binding consensus motif, making it difficult to be targeted in structure (55). Given that SPOP upregulates RIPK1 protein levels, rendering cancer cells more prone to apoptosis, RIPK1 could be an effective target for tumors with overexpressed SPOP like RCC. IAPs are important components of TNF-RSC and mediate K63 ubiquitination of RIPK1. Smac mimetics can neutralize X-linked inhibitor of apoptosis protein (XIAP), cIAP1, and cIAP2 and induce RIPK1-dependent apoptosis in a variety of tumors (56, 57). To date, no studies have investigated whether SM164 can enhance the antitumor efficacy of TKIs. In this study, we selected 3 types of tumors characterized by aberrantly high expression of SPOP and RIPK1 to evaluate the effects of combination therapy. It was found that the efficacy of SM164 in combination with sunitinib was significantly better than sunitinib monotherapy, and this sensitizing effect was dependent on the expression level of RIPK1. Previous studies have demonstrated the potential of SM164 to sensitize tumors to radiotherapy and chemotherapy. One study reported that SM164 enhanced the efficacy of gemcitabine in pancreatic cancer (58). Yang et al. demonstrated that SM164 enhanced the efficacy of radiotherapy in HNSC through the activation of caspases (59). Another study on osteosarcoma showed that SM164 combined with adriamycin treatment can downregulate XIAP expression, thereby enhancing the antitumor activity of adriamycin (60). Our findings suggest that a RIPK1 expression–based targeted combination therapy may help identify potential beneficiaries in patients with advanced tumors.

In conclusion, our research elucidates that SPOP, as a key regulator of the TNF-α–mediated RCD pathway, differentially regulates apoptosis and necroptosis. SPOP mediates RIPK3 ubiquitination and degradation, inhibiting necrosome formation and thereby preventing necrosis. Additionally, SPOP interacts with RIPK1 independently of its E3 ligase activity, protecting RIPK1 from ubiquitination and degradation, thus increasing RIPK1 expression and sensitizing cells to RIPK1-dependent apoptosis. Moreover, pancancer analysis has shown that the SPOP/RIPK1/RIPK3 axis is dysregulated in various tumors. Based on this, we developed a combination therapy of SM164 and sunitinib for tumor types with high expression of SPOP/RIPK1, and the efficacy was significantly better than that of sunitinib monotherapy. These results indicate the clinical potential of combining TKI with Smac mimetics in SPOP/RIPK1/RIPK3 dysfunction tumors represented by RCC.

This study has certain limitations that should be addressed in future work. Although the regulation of cell death markers by SPOP is significant at the molecular level, the observed effects of SPOP on intracellular cell death pathways are modest. The specific binding sites of SPOP and RIPK1, as well as the specific ubiquitin ligases involved in the regulation of RIPK1 ubiquitination, have not been deeply resolved. Therefore, it is necessary to further investigate the binding mechanism in the future by designing truncating mutant plasmids or site-specific mutations. In addition, we did not validate the expression of SPOP, RIPK1, and RIPK3 in clinical samples of LIHC, LUAD, COAD, and BRCA.

## Methods

### Sex as a biological variable.

Sex was not considered as a variable. Our study examined male mice because male animals exhibited less variability in phenotype. It is unknown whether the findings are relevant for female mice.

### Cell culture and reagents.

RCC cell lines (786-O, A498, Caki-1), LIHC cell lines (Huh7, HepG2), LUSC cell lines (H199, A549), COAD cell lines (HCT116, LOVO), BRCA cell lines (MDA-MB-231, MCF7), and the normal kidney cell lines HEK293T and HK-2 were purchased from American Type Culture Collection. *SPOP^+/+^* and *SPOP^–/–^* MEFs derived from *SPOP^+/+^* and *SPOP^–/–^* mice (17) were obtained from Wenyi Wei (Harvard Medical School, Boston, Massachusetts, USA). *RIPK3^+/+^* and *RIPK3^–/–^* MEFs were obtained from Junying Yuan (Harvard Medical School, Boston, Massachusetts, USA). Cells were cultured in DMEM with 10% FBS and maintained in an incubator with 5% CO_2_ at 37°C. Lentivirus packaging and cell transfection were conducted as indicated previously (53).

The reagent and concentration used in the experiment are as follows: recombinant mouse TNF-α (Cell sciences, CRT192C, 100 ng/mL), SM-164 (MedChemExpress [MCE], HY-15989, 50 nM), Nec-1s (Selleck, S8641, 20 μM), 5z7 (Sigma, 09890, 500 nM), zVAD.fmk (Sigma, V116, 25 μM), cycloheximide (Sigma, C-6255, 1 μM), and GSK872 (MilliporeSigma, 530389, 10 μM).

The reagent combinations used in the experiment are as follows: T5z7 was used to induce apoptosis; TS was used to induce apoptosis; TSZ was used to induce necroptosis; and T5z7Z was used to induce necroptosis.

### Patients and tissue samples.

Tumor tissues of RCC, as well as adjacent normal tissues, were obtained from patients who underwent nephrectomy at the Department of Urology, Union Hospital (Wuhan, China). Following removal, all samples were stored in liquid nitrogen. The patients gave their informed consent, and the Tongji Medical College ethics committee approved the procedure.

### Plasmids and sgRNAs.

Flag-RIPK3 and shRIPK1 were obtained from Junying Yuan (Boston, Harvard Medical School, Boston, Massachusetts, USA). His-ub and SPOP-related constructs were obtained from Wenyi Wei (Harvard Medical School, Boston, Massachusetts, USA). Related constructions will be provided in detail upon request. The sgSPOP sequences were as follows: sgSPOP-1-F (5′-CACCGTGTTTGCGAGTAAACCCGAA-3′), sgSPOP-1-R (5′-AAACTTCGGGTTTACTCGCAAACAC-3′), sgSPOP-2-F (5′-CACCGTGCCGGTTGGCAGATGAGTT-3′), and sgSPOP-2-R (5′-AAACAACTCATCTGCCAACCGGCAC-3′).

### Immunoblots and immunoprecipitation.

Total protein was extracted from cells or tissues after lysis in RIPA buffer with protease inhibitors and phosphatase inhibitors (Servicebio). SDS-PAGE was used to separate a similar amount of each lysate, which was then immunoblotted with the indicated antibodies. In immunoprecipitation, cell lysate was incubated with the indicated antibody overnight at 4°C, followed by 1-hour rotation with Protein A/G Magnetic Beads (MCE). Immunoprecipitants were washed for 4 times and boiled in 5× SDS sample buffer before immunoblotting. For in vivo ubiquitination assays, HEK293T cells were transfected with the designed constructs 36 hours before being incubated for 6 hours with 20 μM MG132. Following assays were conducted as described previously (17). All antibodies (Supplemental Table 1) were used at a 1:1,000 dilution for Western blots.

### Quantitative reverse transcription PCR.

TRIzol reagent (Takara) was utilized to extract total RNA from cells, followed by assessment of RNA concentration and purity using a Bio-Tek Epoch spectrophotometer. To generate cDNA, the RNA was reverse-transcribed using the HiScript III 1st Strand cDNA Synthesis Kit (Vazyme) in accordance with the manufacturer’s instructions. The sequences of primers employed in this study (TSINGKE) are provided in Supplemental Table 2.

### MTT assay.

Cell viability was assessed using the MTT assay. Briefly, indicated cells like MEFs and 786-O were seeded in 96-well plates at a density of 10,000 cells/well in complete DMEM and allowed to adhere overnight. Cells were then treated with different reagents (see Figure 1 and corresponding legend) for 4 hours. Following treatment, MTT solution (Sigma, catalog M2128) was added to each well to a final concentration of 0.5 mg/mL and incubated at 37°C for 2 hours. The formazan crystals formed were subsequently dissolved in DMSO by gentle agitation. The absorbance was measured at a wavelength of 570 nm using a microplate reader. Cell viability was calculated as a percentage relative to the untreated control wells. Experiments were performed in triplicate and repeated 3 times independently. Data are presented as mean ± SD.

### CCK-8 assay.

CCK-8 assay (MCE, catalog HY-K0301) was used to evaluate cell proliferation/viability according to the manufacturer’s protocol. Briefly, indicated cells were seeded in 96-well plates and treated with reagents for 4 to 6 hours. At the end of the treatment period, 10 μL of the CCK-8 solution was added directly to each well containing 90 μL of culture medium. The plates were then incubated at 37°C in the dark for 2 hours. Absorbance was measured at 450 nm using a microplate reader. Cell viability was calculated and data presented as described in *MTT assay*.

### IHC assay.

Standard IHC was performed on formalin-fixed, paraffin-embedded tissue sections prepared as described in Supplemental Methods. Sections underwent antigen retrieval using heat-induced epitope retrieval in PBS for 10 minutes in a steamer. Endogenous peroxidase activity was blocked by incubating sections in 3% H_2_O_2_ solution for 15 minutes at room temperature (RT). Nonspecific binding was blocked using 5% BSA in PBS for 10 minutes at RT. Sections were then incubated overnight at 4°C with primary antibodies (listed in Supplemental Table 2) diluted 1:1,000 to 1:2,000 in antibody diluent. After washing in PBS (3 times for 5 minutes each), sections were incubated with secondary antibody (listed in Supplemental Table 2) for 60 minutes at RT. Immunoreactivity was visualized using DAB chromogen kit (Proteintech, catalog PR30010). Sections were then dehydrated, cleared, and mounted as described for H&E staining. Appropriate positive and negative controls (primary antibody omitted or replaced with nonimmune serum/blocking solution) were included in each staining run. Slides were evaluated using light microscopy. Percentage of positive cells was semiquantitatively assessed and scored by 2 independent observers blinded to the experimental groups using Intensity × % positive cells (61).

### In vivo RCC subcutaneous xenograft assay.

BALB/c nude mice (6 weeks old, male, HFK Bio-technology) were inoculated in the armpit with 786-O cells. Every other day, the mice were treated with either single-agent sunitinib or sunitinib plus SM164. Sunitinib was given orally via gavage needle at a dose of 40 mg/kg, and SM164 was intraperitoneally injected at 5 mg/kg. The tumor was measured every 5 days. The mice were sacrificed after 35 days, and the tumor weight was then measured. All animal experiments were performed as indicated previously (62).

### Bioinformatics analysis.

The pancancer dataset was downloaded from the UCSC database under the instructions of Sangerbox (63), from which we further extracted the expression data of RIPK1, RIPK3, and SPOP gene in each sample. Next, we excluded the samples with gene expression level of 0 and restricted the samples to primary tumors and normal tissues. Those tumor types with fewer than 3 samples were then excluded. Each expression value was further log2(TPM+1) transformed for analysis. R (version 3.2.0) was used to explore the expression differences of genes and the correlation among genes.

### Statistics.

The mean ± SD is used to express the data. The significance of differences was revealed based on unpaired or paired 2-tailed Student’s *t* test for 2 independent groups, Wilcoxon’s rank sum and signed rank tests for 2 groups in multiple samples, Kruskal’s test for multiple groups, and 1-way or 2-way ANOVA with Tukey’s post hoc test. The correlation analysis was conducted using Pearson’s correlation coefficient. *P* < 0.05 was considered statistically significant. Each experiment in vitro was performed in triplicate.

### Study approval.

This study was approved by the Tongji Medical College ethics committee: No. [2021] IEC (080). Written informed consent was obtained from the patients participating in the study. All animal experiments were approved by the Animal Ethics Committee of Tongji Medical College (No. 0136).

### Data availability.

The datasets used and analyzed during the current study are available in the Supporting Data Values XLS file.

## Author contributions

LL, ZZ, YY, and CY designed and performed the study. WX, HR, YZ, and QM analyzed the data and performed the literature search. XZ, ZZ, WX, and LL contributed the essential reagents and supervised the research. YY, LL, and CY wrote the paper. All authors read and approved the paper.

## Funding support

National Natural Science Foundation of China (Grant Nos. 82102787, 82372845).

Investigator Initiation Fund Project of Fujian Medical University Union Hospital (Grant No. 2024XH042).

Shenzhen Medical Research Fund (Grant No. B2302054).

Joint Funds for the innovation of science and Technology, Fujian province (Grant No. 2060304).

Fujian Provincial Natural Science Foundation of China (Grant No. 2025J01137).

## Supplementary Material

Supplemental data

Unedited blot and gel images

Supporting data values

## Version 1 10/22/2025

Electronic publication
