# Supplementary material for: SPOP mediates apoptosis and protects against necroptosis by regulating ubiquitination of RIPK1 and RIPK3
Source: JCI Insight. 2025 Oct 22;10(20):e180655. doi: 10.1172/jci.insight.180655 (PMC12581659; doi:10.1172/jci.insight.180655)
Supplement: Supplemental data [file jciinsight-10-180655-s028.pdf]

Supplementary Information

1. Supplemental Methods

Flow Cytometric Analysis

Apoptosis was assessed by flow cytometry using Annexin V-FITC and propidium iodide (PI) double staining. Mouse Embryonic Fibroblasts (MEFs) were seeded in 6-well plates at a density of 2×10^5 cells/well in complete DMEM medium. After 24 hours of adherence, cells were treated under indicated conditions for 24 hours at 37°C in a 5% CO_2 incubato. Cells were then harvested, washed twice with cold PBS, and resuspended in 100 μL of 1× Annexin V Binding Buffer (Annexin V-FITC Apoptosis Detection Kit, BD Biosciences, Cat# 556547). Subsequently, 5 μL of Annexin V-FITC and 5 μL of PI working solution were added to the cell suspension, followed by gentle vortexing and incubation in the dark at 4°C for 15 minutes. Stained cells were analyzed within 1 hour using a flow cytometer, acquiring at least 10,000 events per sample. Excitation was performed with a 488-nm laser; Annexin V-FITC fluorescence was detected in the 530/30 nm bandpass filter channel, and PI fluorescence was detected in the >670 nm longpass filter channel. Data were analyzed using FlowJo (BD Biosciences). Cells were gated based on forward scatter (FSC-A) versus side scatter (SSC-A) to exclude debris, followed by FSC-H versus FSC-A gating to exclude doublets. Apoptotic cells were defined as follows: Annexin V^-/PI^- (viable), Annexin V^+/PI^- (early apoptotic), Annexin V^+/PI^+ (late apoptotic/necrorotic). Results are reported as the percentage of total apoptotic cells (Annexin V^+ cells). Each experiment was performed in triplicate and repeated independently at least three times. Statistical analysis was performed using one-way ANOVA with Tukey's post hoc test in GraphPad Prism 9.0.

Protein-Protein Docking Prediction

Obtain protein information and structures from the UniProt website (https://www.uniprot.org/uniprotkb). Import the retrieved protein structures into Discovery Studio 2019 for structural optimization. This process primarily includes removing water molecules, adding hydrogen atoms, assigning charges, completing amino acid residues, and reconstructing side chains. After obtaining the optimized protein structures, import them into GRAMM, designating the receptor and ligand proteins respectively for docking. Upon completion of the docking, use PyMOL 3.1 for visualization.

Hematoxylin and Eosin (H&E) Staining

Tissue samples tumor xenografts were fixed in 4% paraformaldehyde for 12 to 16 hours, processed through graded ethanol series, embedded in paraffin, and sectioned at 4 μm thickness using a microtome. Sections were mounted on glass slides, deparaffinized in xylene, and rehydrated through a graded ethanol series to distilled water. Sections were stained with hematoxylin solution for 5 minutes, rinsed in running tap water, differentiated briefly in 1% acid alcohol, rinsed again, and then

immersed in bluing solution 1% ammonia water. Sections were counterstained with 0.5% eosin solution for 2 minutes. Following dehydration through graded ethanol, clearing in xylene, and mounting with a cover slip, sections were visualized and imaged under a light microscope. Representative images were captured at 200x and 400x magnification. Evaluation of tissue morphology was performed by a board-certified pathologist in a blinded manner.

2. Supplementary Tables

Table S1. Antibodies used in this study.

Company	Antibody (Catalog Number)
Cell Signaling	Anti-PARP (9532), anti-Cleaved-Caspase3 (9664), anti-RIPK1 (3493) anti-p-RIPK1 (65746 & 31122), anti-RIPK3 (96702), anti-p-RIPK3 (91702), anti-MLKL (37705), anti-p-MLKL (37333), anti-P38 (9212), anti-p-P38 (9211), anti-JNK (9252), anti-p-JNK (9255), anti-IκB (4814), anti-p-IκB (5209), anti-ERK1/2 (4695), anti-p-ERK1/2 (4370)
Santa Cruz	Anti-TRIM24/TIF1α (C-4), anti-HA (sc-805), anti-β-actin (81178)
Proteintech	Anti-SPOP (16750-1-AP)
Sigma	Anti-Flag antibody (F-3165, clone M2), anti-Flag agarose beads (A-2220), peroxidase-conjugated anti-mouse secondary antibody (A-4416) and peroxidase-conjugated anti-rabbit secondary antibody (A-4914)
BD Bioscienses	Anti-RIPK1 (610459)

Table S2. Primes used in this study.

Primers	Sequence
GAPDH FP	5'- GAGTCAACGGATTTGGTCGT -3'
GAPDH RP	5'- GACAAGCTTCCCGTTCTCAG -3'
RIPK3 FP	5'- ATGTCGTGCGTCAAGTTATGG -3'
RIPK3 RP	5'- CGTAGCCCCACTTCCTATGTTG -3'

3. Supplementary Figure and Figure Legends

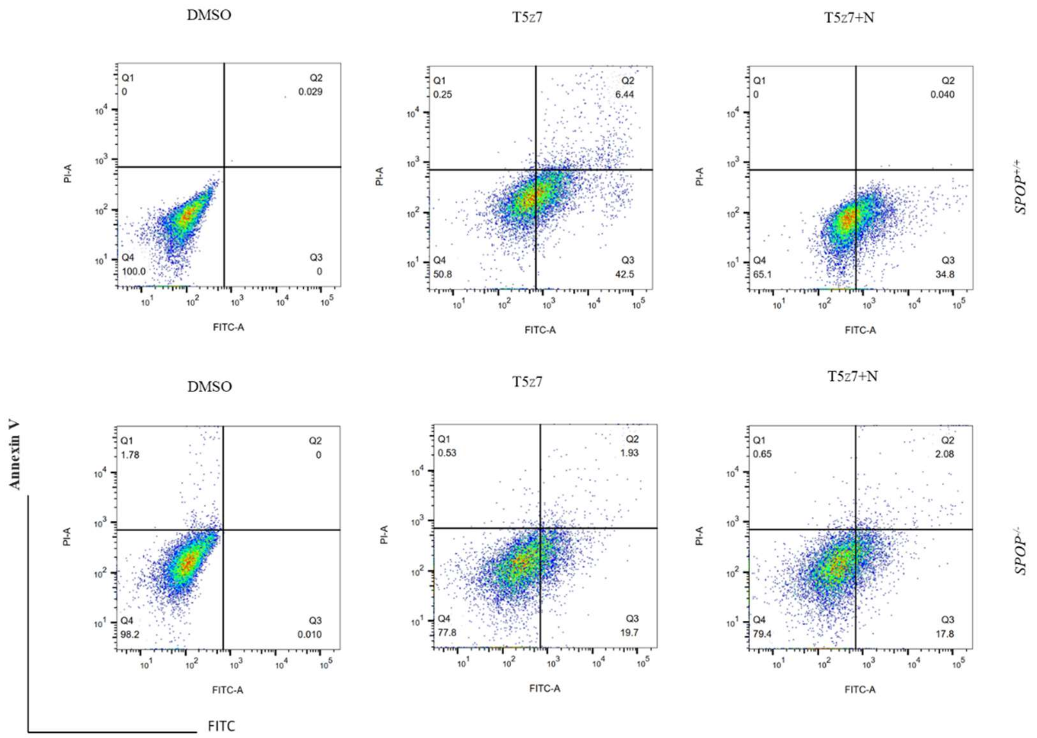

Figure S1. Flow assays to determine the sensitivity of SPOP to MEF cells to TNFα + 5z7 (T5z7) -mediated apoptosis. *SPOP*$^{+/+}$ and *SPOP*$^{-/-}$ MEFs cells pretreated with DMSO or Nec-1s for 1h and then treated with DMSO or T5z7 or T5z7+Nec-1s for 6h, MEF apoptosis was then measured by flow cytometry.

A

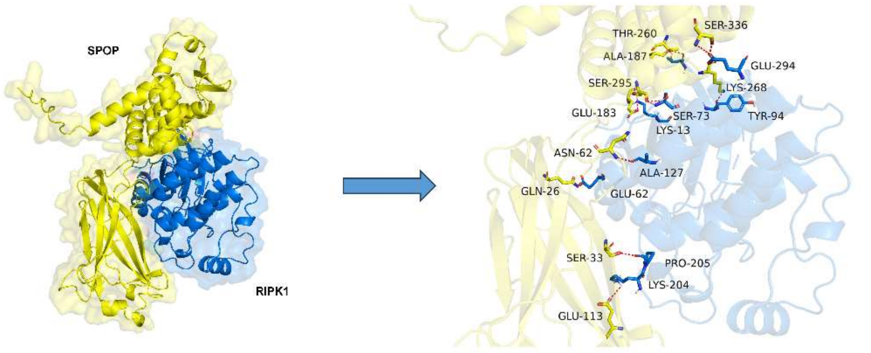

B

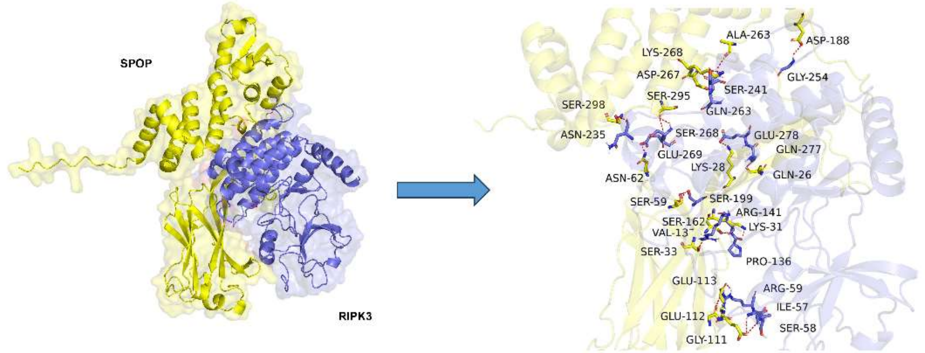

Figure S2. Prediction of protein-protein docking of SPOP and RIPK1 or RIPK3. A) Molecular docking model of SPOP-RIPK1 interaction. B) Molecular docking model of SPOP-RIPK3 interaction. SPOP is shown in yellow, RIPK1 in blue, RIPk3 in purple, with dashed lines indicating hydrogen bonds.

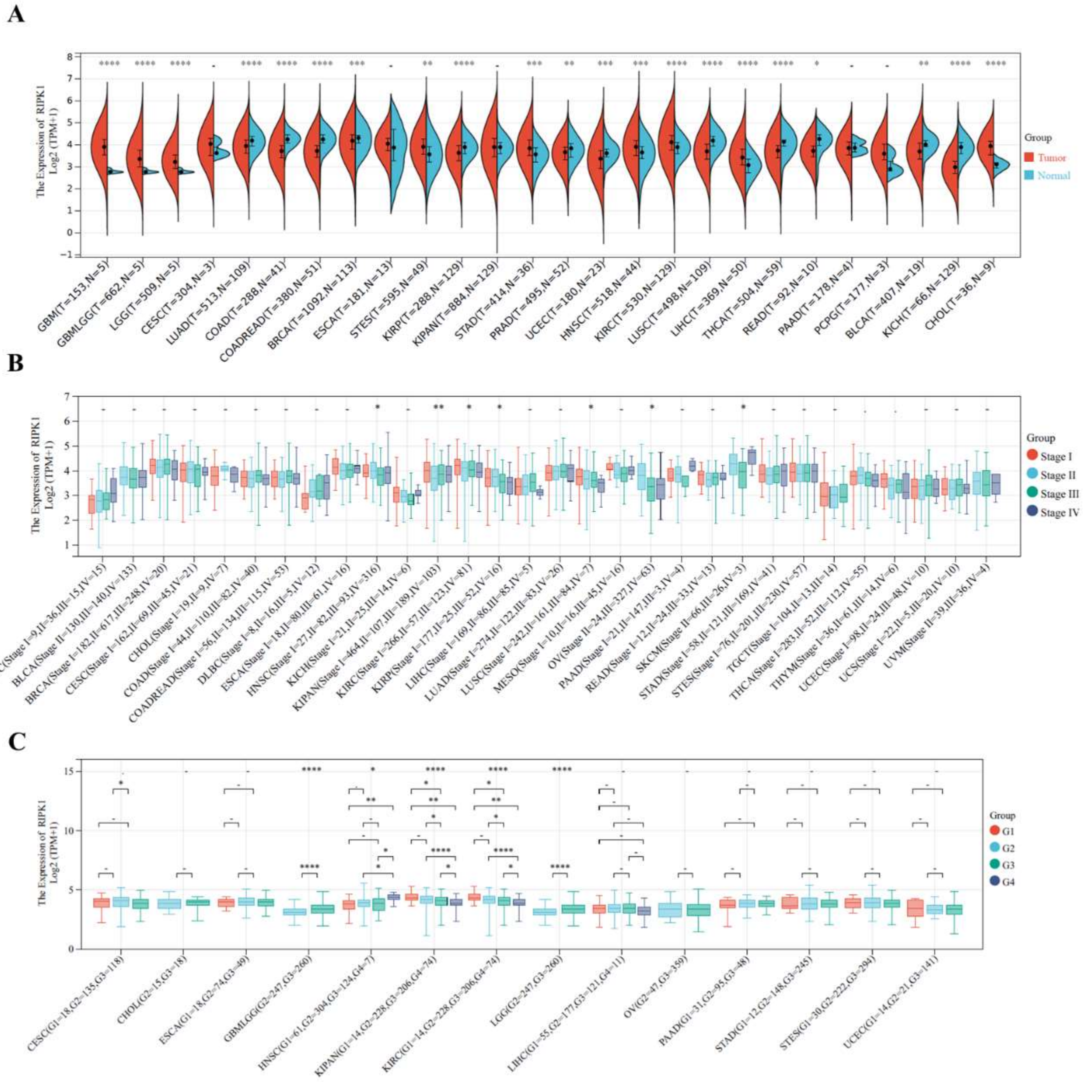

Figure S3. Pan-cancer analysis of RIPK1. A) Pan-cancer analysis of RIPK1 expression in 26 kinds of tumors and corresponding normal tissues after procedural exclusion. **B-C)** Pan-cancer analysis of RIPK1 expression among different clinical stages in 30 kinds of tumors and different pathological grades in 14 kinds of tumors after procedural exclusion.

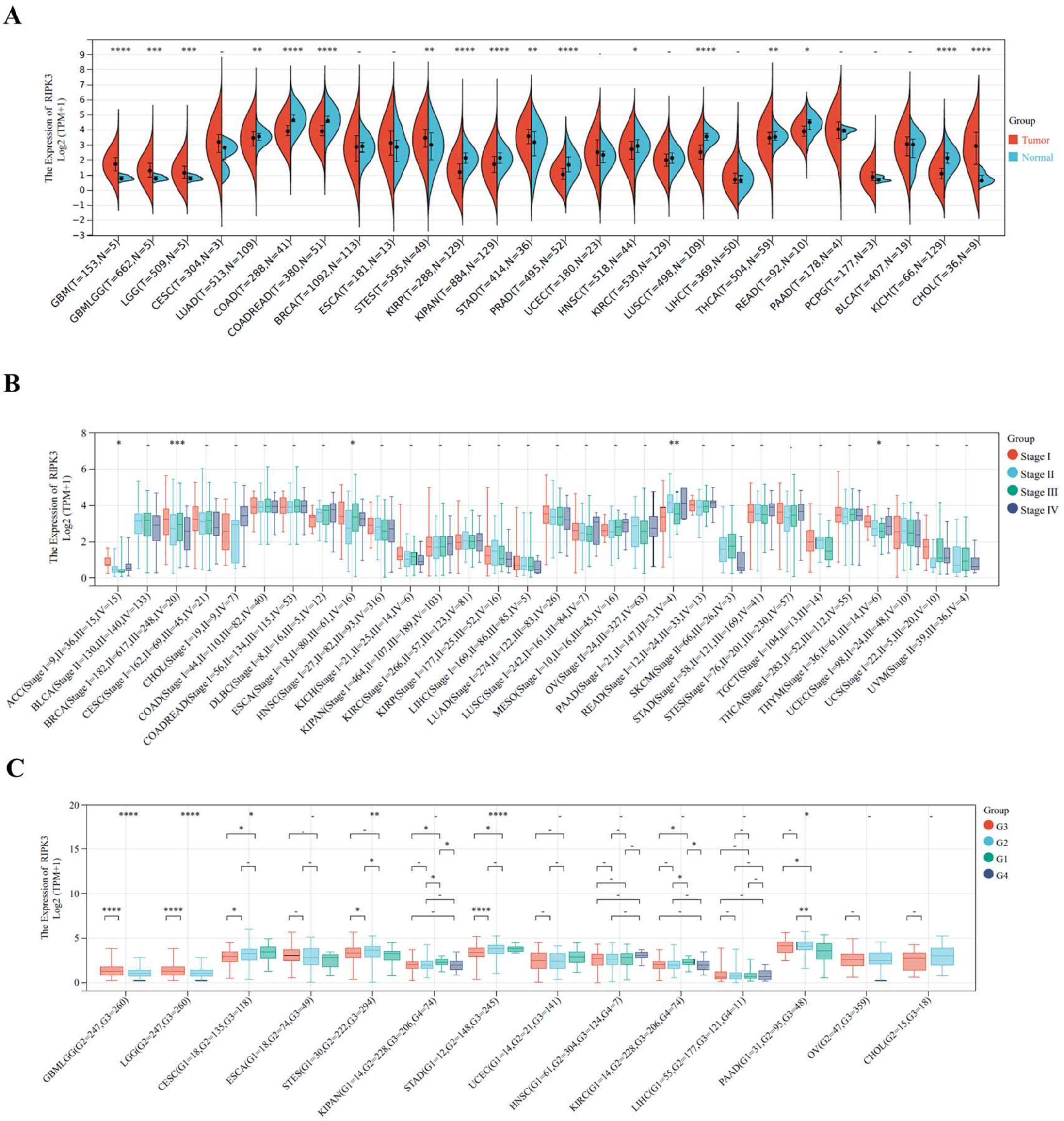

Figure S4. Pan-cancer analysis of RIPK3. A) Pan-cancer analysis of RIPK3 expression in 26 kinds of tumors and corresponding normal tissues after procedural exclusion. **B-C)** Pan-cancer analysis of RIPK3 expression among different clinical stages in 30 kinds of tumors and different pathological grades in 14 kinds of tumors after procedural exclusion.
