# Supplementary material for: SPOP mediates apoptosis and protects against necroptosis by regulating ubiquitination of RIPK1 and RIPK3
Source: JCI Insight. 2025 Oct 22;10(20):e180655. doi: 10.1172/jci.insight.180655 (PMC12581659; doi:10.1172/jci.insight.180655)
Supplement: Unedited blot and gel images [file jciinsight-10-180655-s029.pdf]

Figure 2A

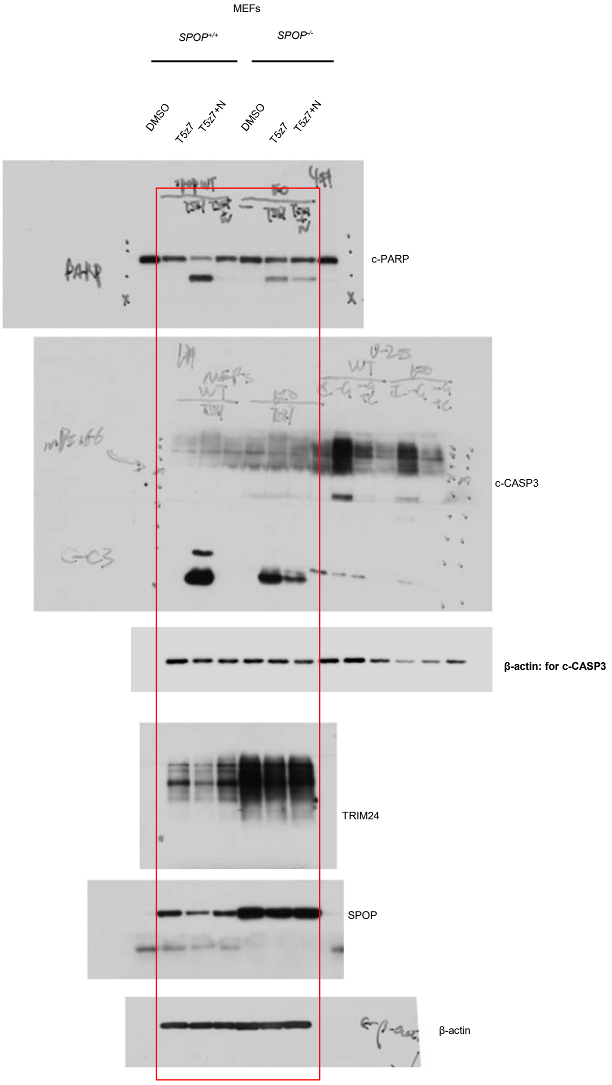

Figure 2B

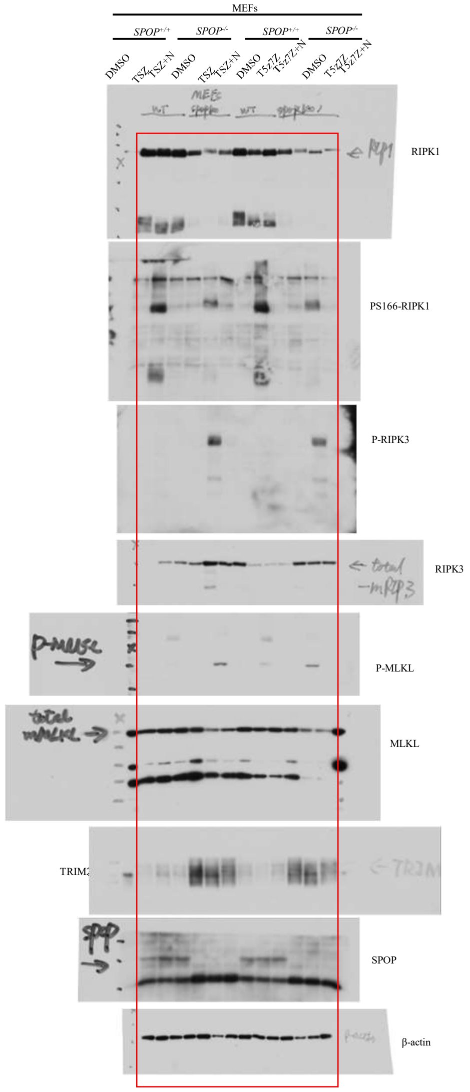

Figure 2C

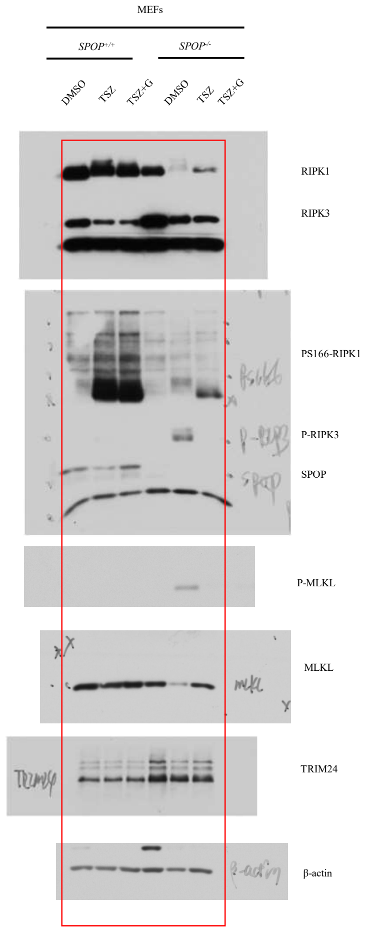

Figure 2D

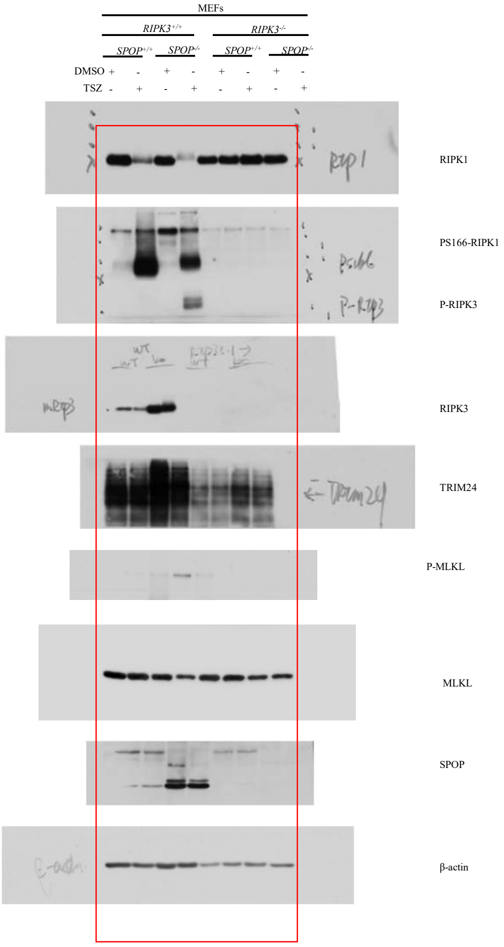

Figure 2F

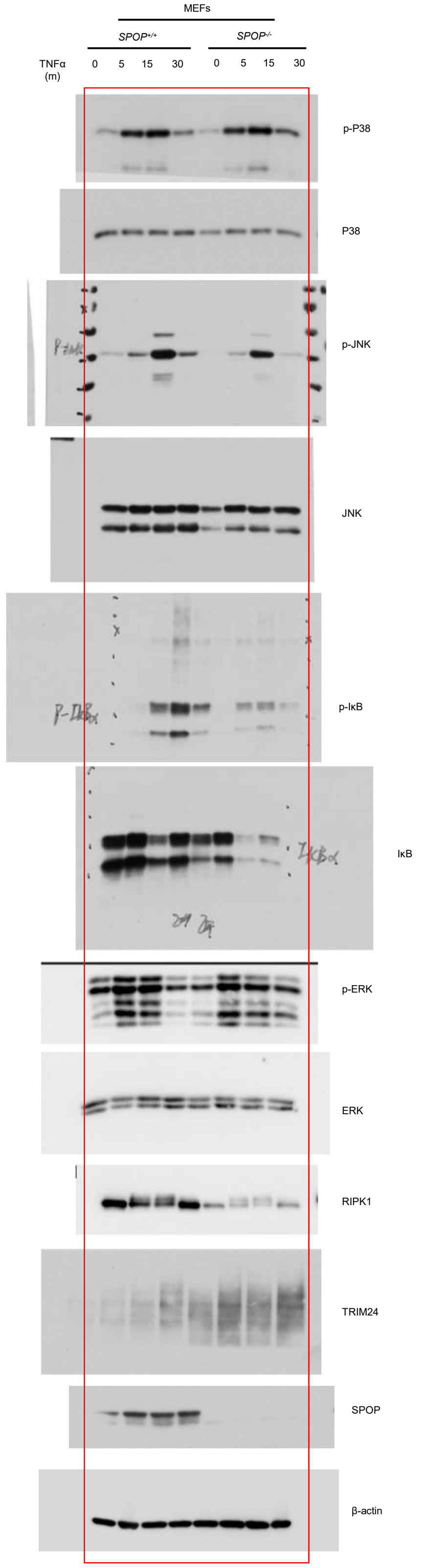

Figure 2G

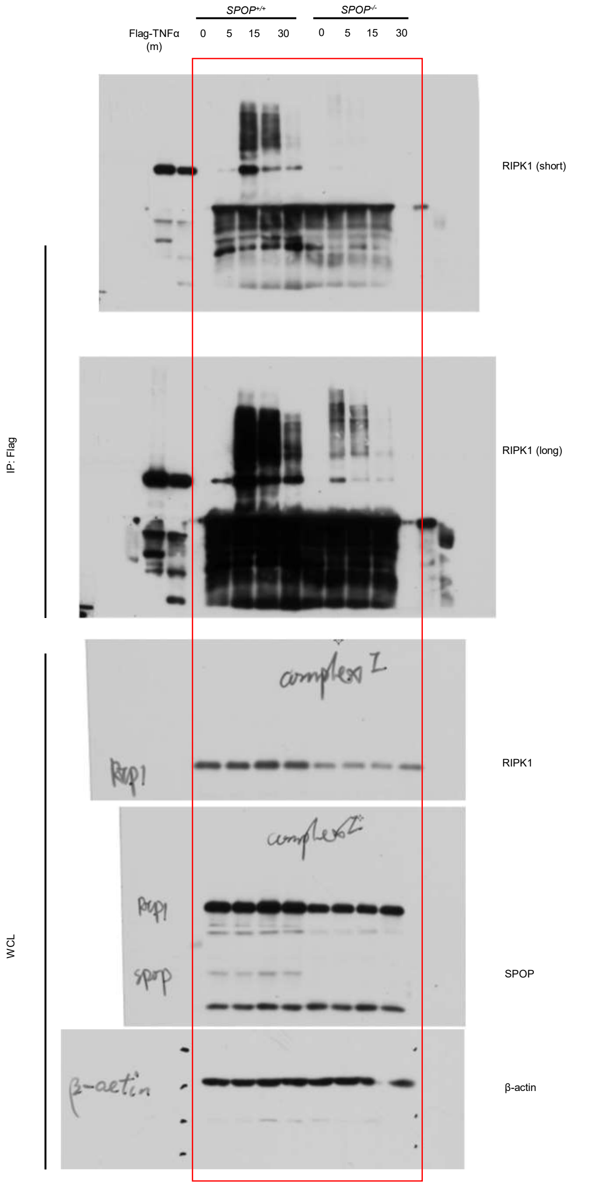

Figure 2H

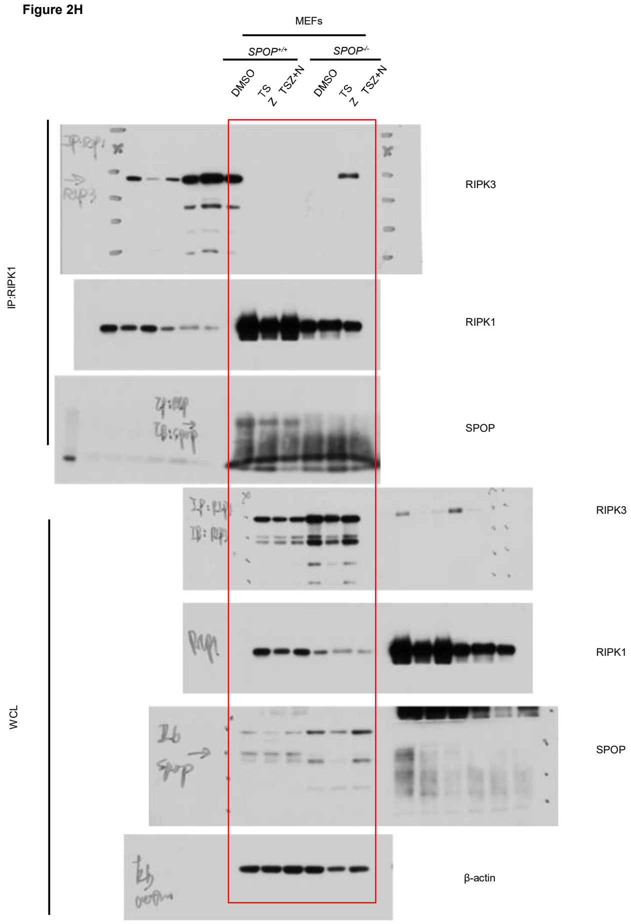

Figure 3A

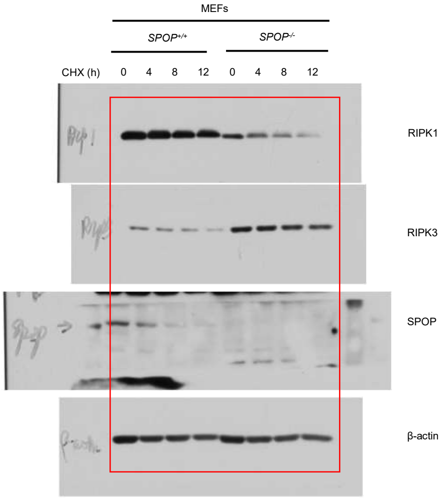

Figure 3B

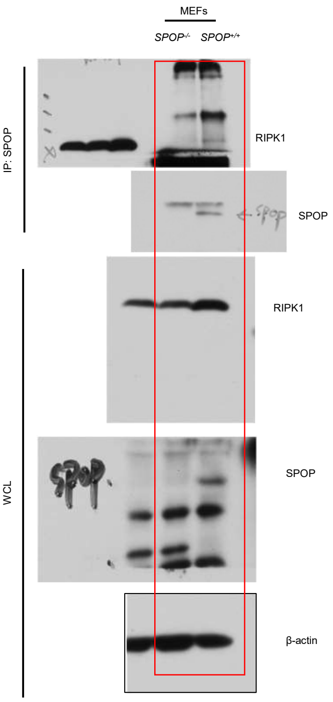

Figure 3C

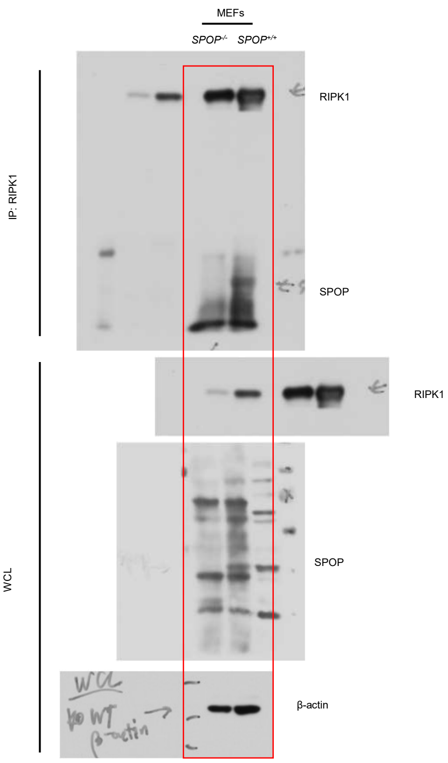

Figure 3D

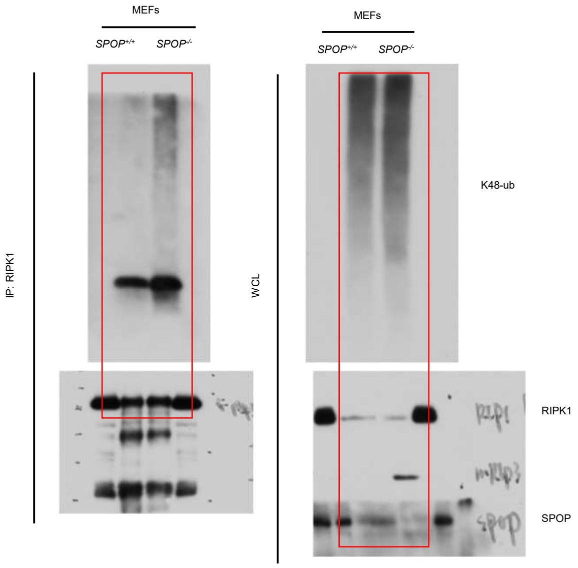

Figure 3E

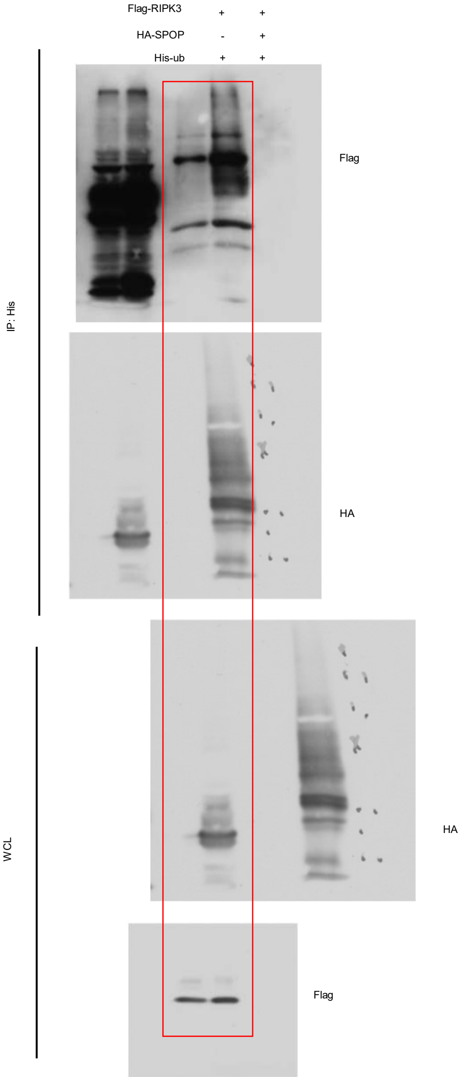

Figure 3G

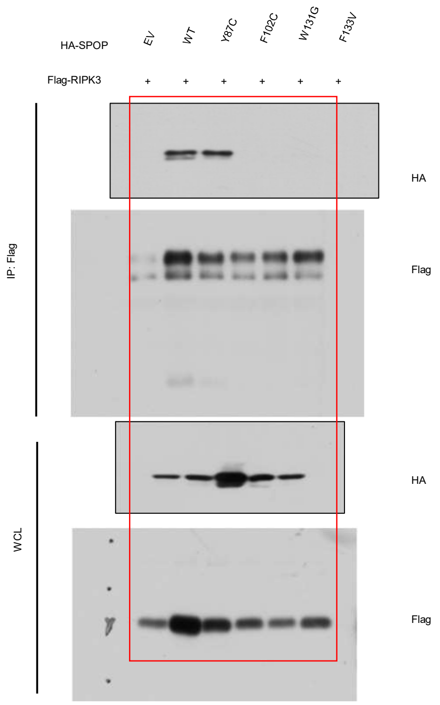

Figure 3H

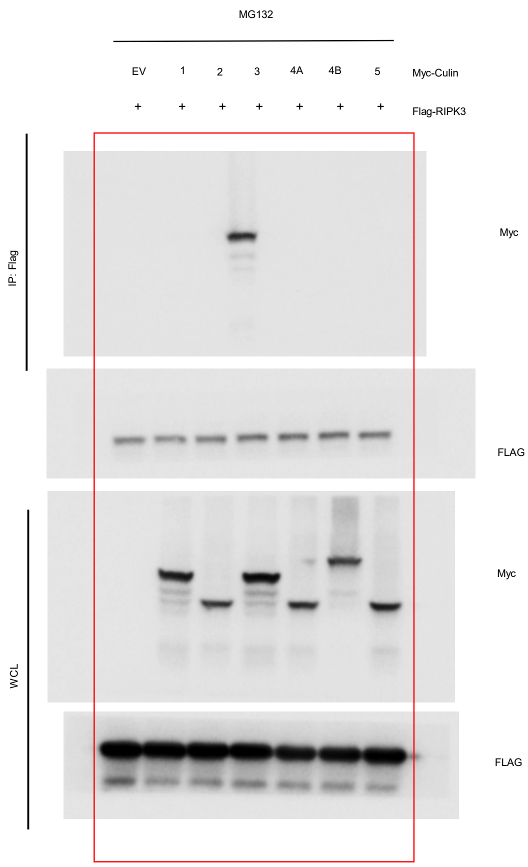

Figure 3I

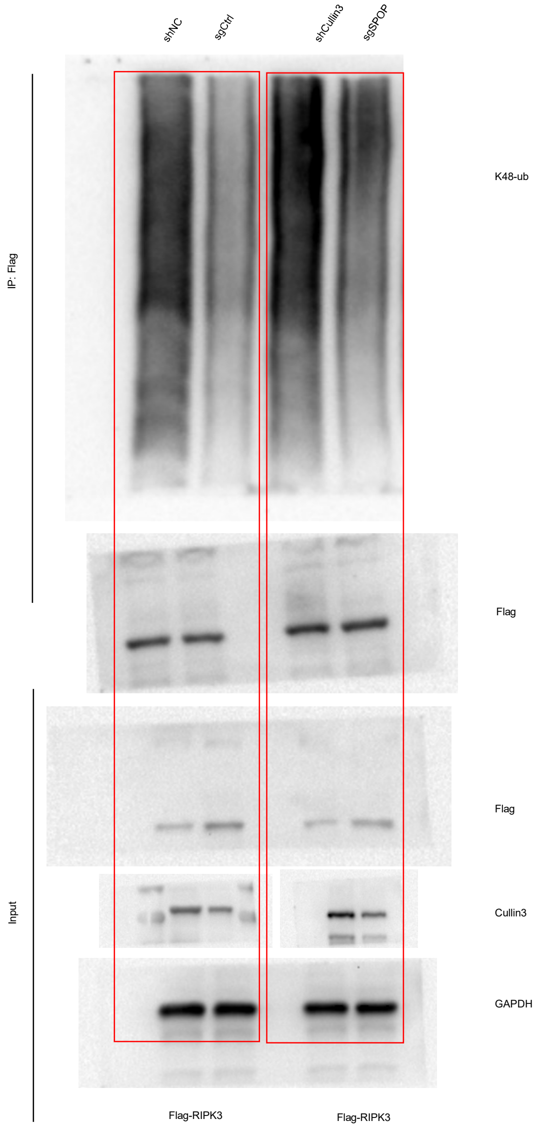

Figure 5C

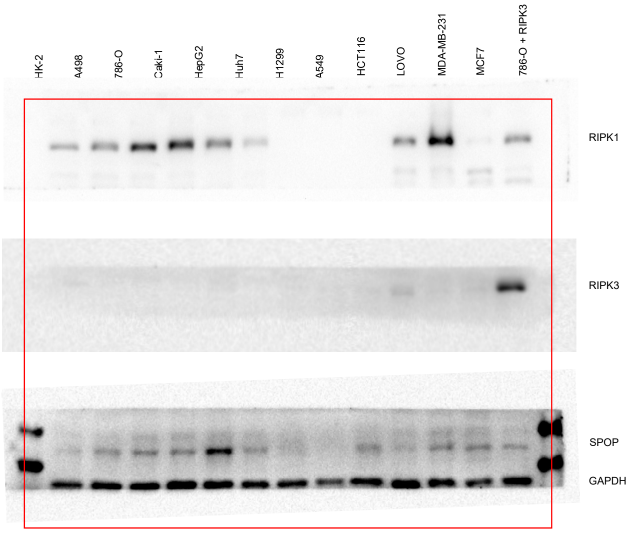

Figure 5E

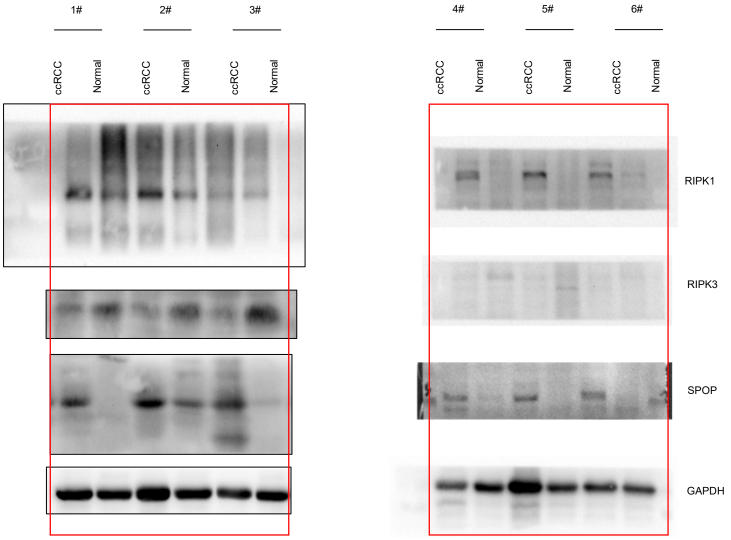

Figure 5F

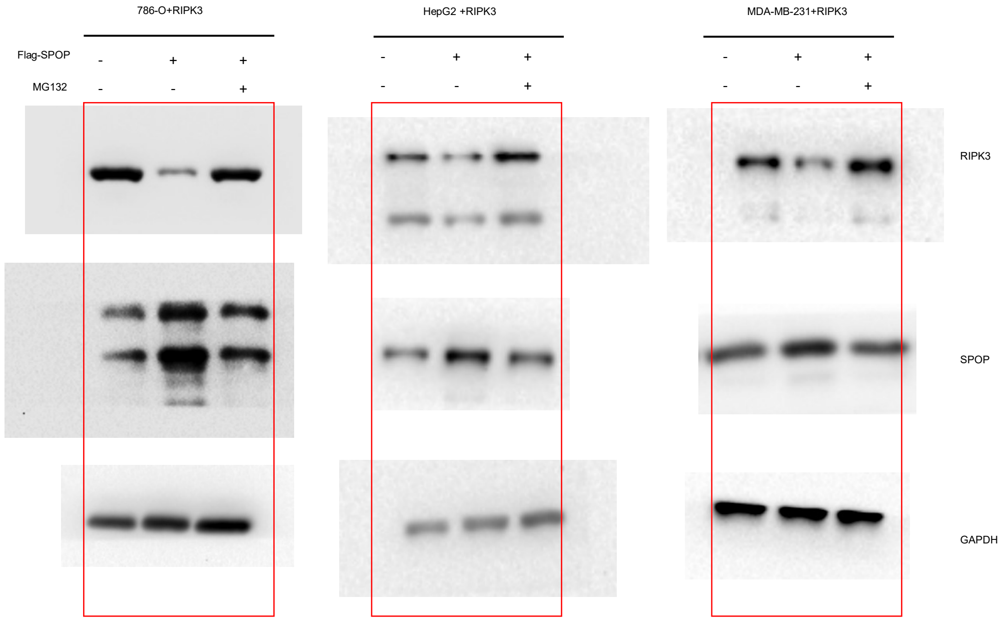

Figure 5G

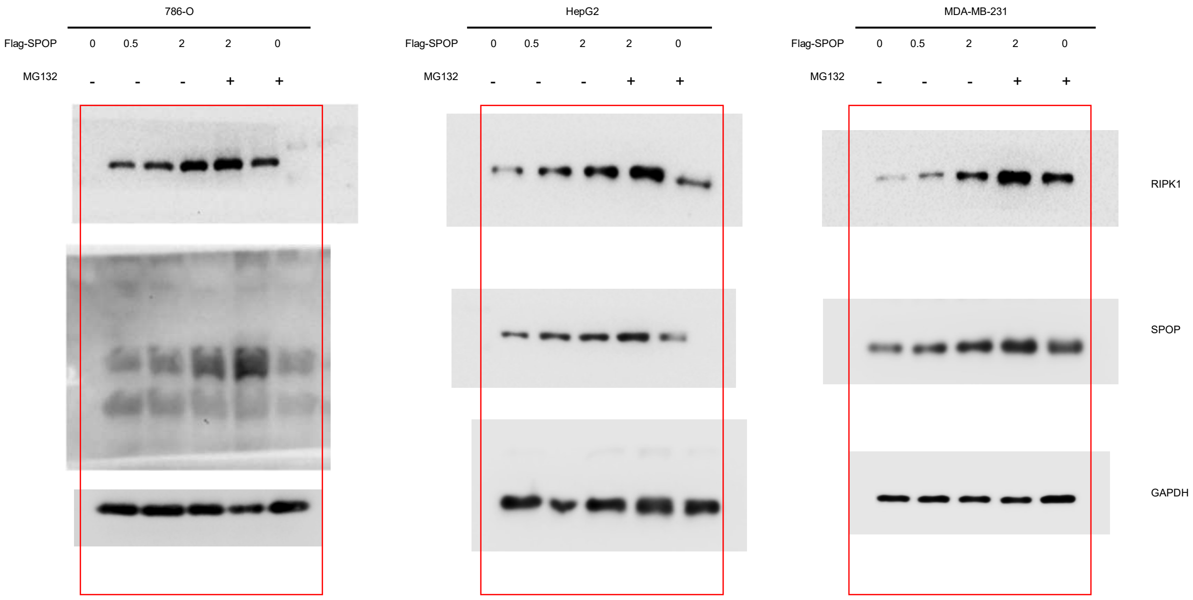

Figure 6A

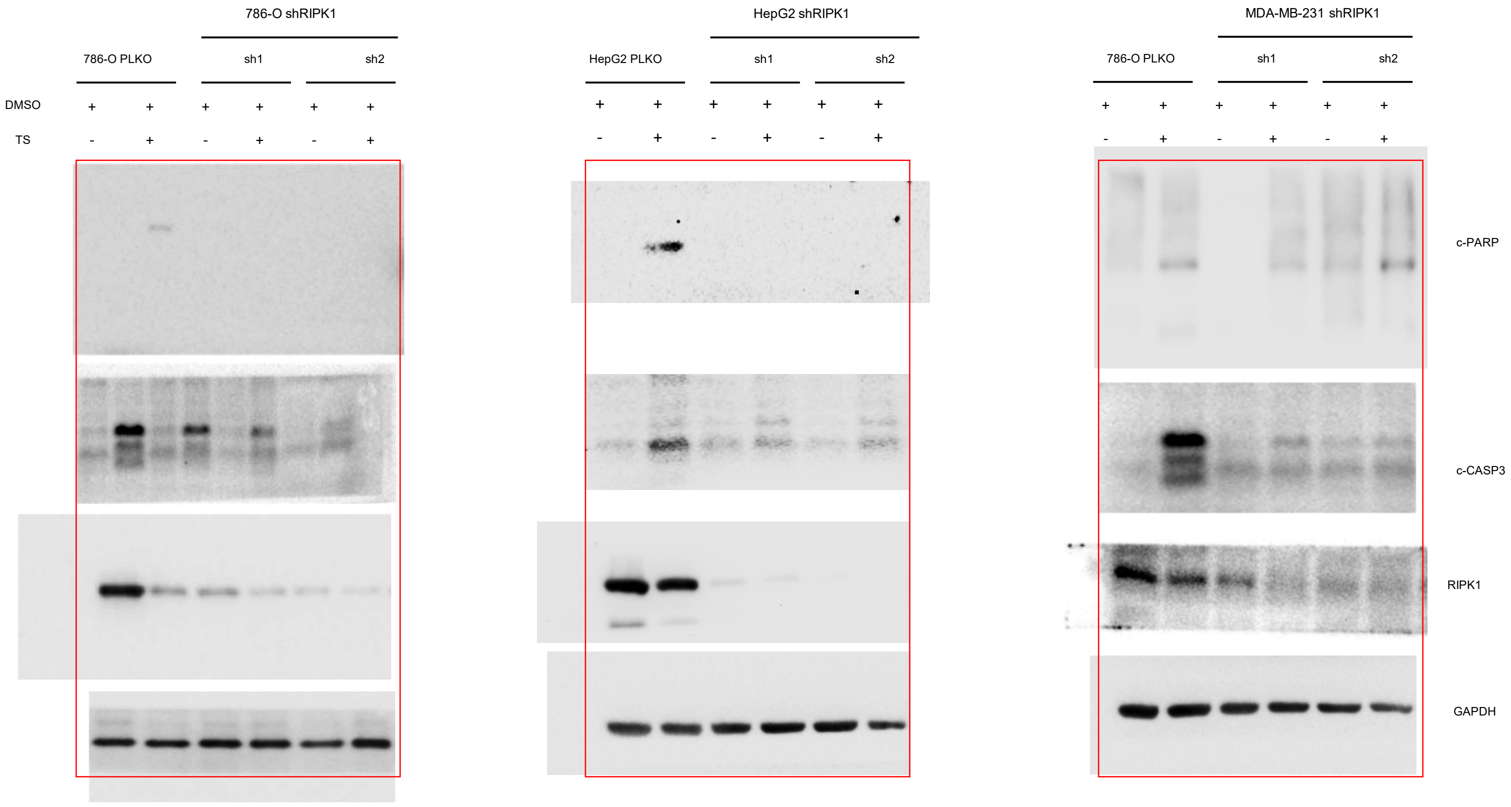

Figure 6B

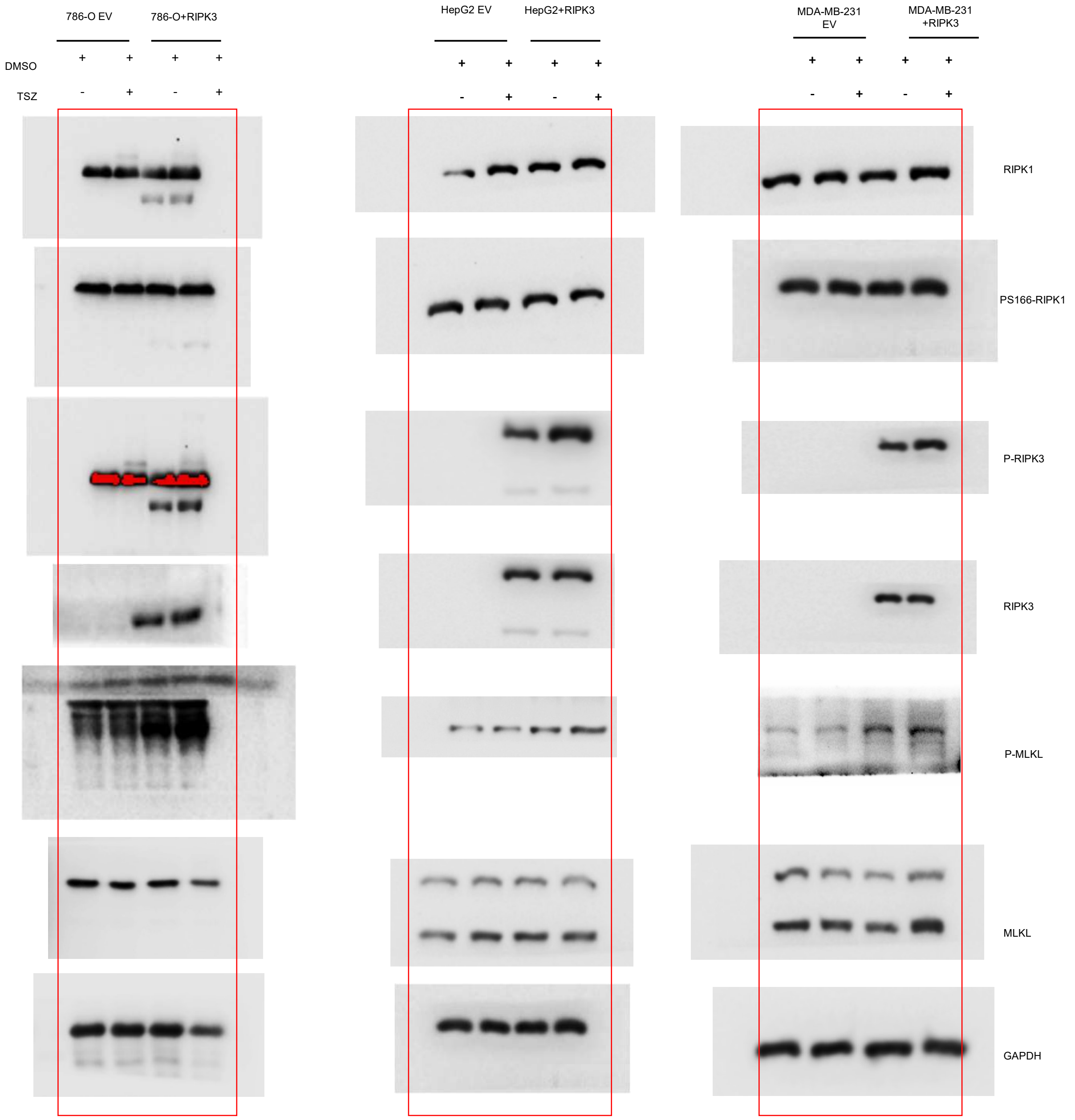

Figure 6G

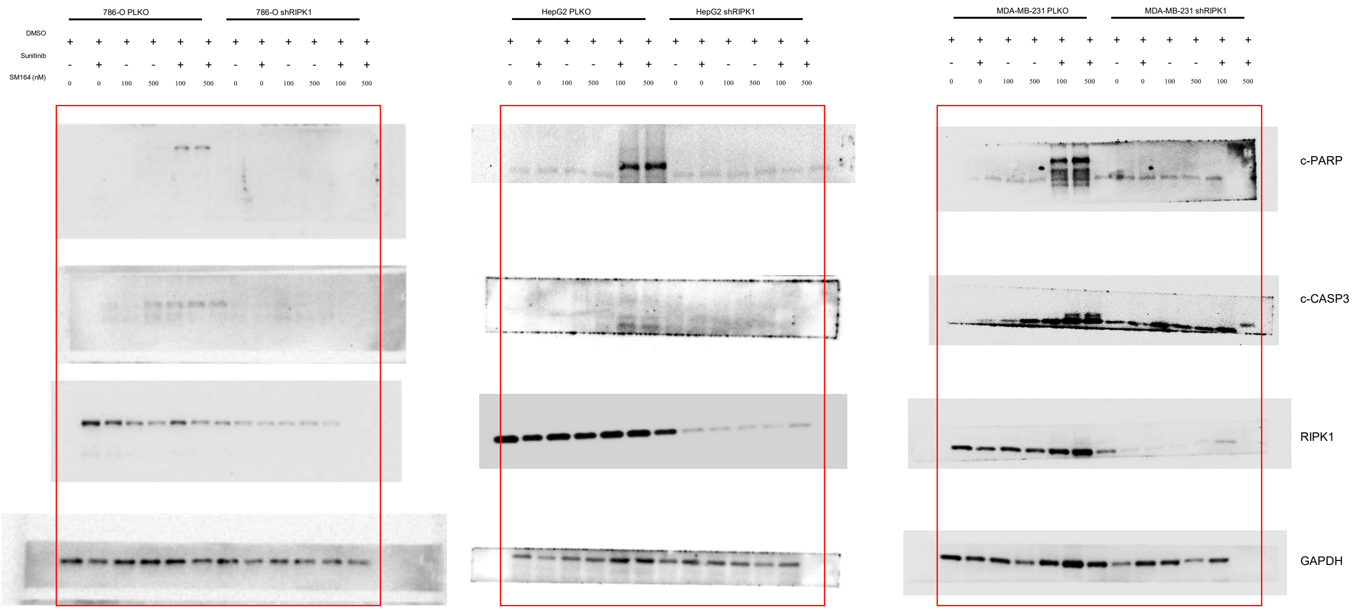
786-O PLKO
786-O shRIPK1
HepG2 PLKO
HepG2 shRIPK1
MDA-MB-231 PLKO
MDA-MB-231 shRIPK1
DMSO
Sunitinib
SM164 (nM)
c-PARP
c-CASP3
RIPK1
GAPDH
